# Prognostic gene screening and experimental validation in renal clear cell carcinoma based on spatial transcriptomics and single-cell sequencing

**DOI:** 10.3389/fimmu.2026.1699883

**Published:** 2026-01-28

**Authors:** Cong Fu, Lin Sun, Tong Zhou, Yanzhi Bi

**Affiliations:** 1 Department of Oncology, Changzhou Cancer (Fourth People’s) Hospital, Changzhou, China; 2 Department of Oncology, Affiliated Hospital of Soochow University, Changzhou, China

**Keywords:** ATP1A1, clear cell renal cell carcinoma, epithelial cells, nomogram, prognostic genes, single-cell RNA sequencing, spatial transcriptomics RNA sequencing

## Abstract

**Background:**

Clear cell renal cell carcinoma (ccRCC) is characterized by high recurrence and metastatic potential, leading to poor clinical outcomes. There is a critical need to identify reliable prognostic biomarkers and therapeutic targets to improve patient stratification and personalized treatment.

**Methods:**

This study integrated single-cell RNA sequencing (scRNA-seq) data and spatial transcriptomics (ST) data to identify prognostic genes and therapeutic targets. Prognostic modeling and validation were performed using The Cancer Genome Atlas (TCGA) and International Cancer Genome Consortium (ICGC) datasets. In addition, functional analyses were conducted to explore the biological roles of candidate genes.

**Results:**

Seven prognostic genes (CYFIP2, MPPED2, HHLA2, ADAM8, ATP1A1, ARC, and MXD3) were identified and used to construct a risk model that stratified patients into high- and low-risk groups. The high-risk group exhibited significantly poorer survival, a finding validated in both TCGA and ICGC datasets. A nomogram incorporating risk score and age improved survival prediction accuracy, with Area Under the Curve (AUC) values of 0.79, 0.75, and 0.78 at 1, 3, and 5 years, respectively. ATP1A1 was highly expressed in endothelial cells and was significantly associated with M1 macrophages; thus, it was selected as a potential therapeutic target. Functional analyses revealed its role in angiogenesis inhibition and M1 macrophage polarization.

**Discussion:**

The risk model and nomogram demonstrate strong prognostic value and may aid in clinical risk stratification for ccRCC. ATP1A1 emerges as a potential therapeutic target, with functional implications in angiogenesis and immune modulation. These findings highlight the clinical relevance of the identified gene signatures and support the development of personalized treatment strategies for ccRCC patients.

## 1 Introduction

Kidney cancer is a lethal malignant tumor of the urogenital system. According to estimates from GLOBOCAN 2022, there were approximately 434,000 new cases of kidney cancer worldwide in 2022 (accounting for 2.2% of all cancer cases), resulting in approximately 156,000 deaths (representing 1.6% of all cancer-related deaths) (1). Nearly 70% of all confirmed adult kidney tumor cases are clear cell renal cell carcinoma (ccRCC), which is distinguished by malignant renal tubular epithelial cells with transparent cytoplasm (2). ccRCC presents with a unique molecular profile characterized by widespread DNA mutations and chromosomal abnormalities, leading to the loss of heterozygosity of tumor-suppressor genes (3). Among these molecular abnormalities, of which chromosomal deletions are a core type, the most frequent chromosomal event in ccRCC is the deletion in the short arm of chromosome 3, which involves the loss of genes in this region, including VHL, PBRM1, SETD2, and BAP1 (3). The frequent inactivation and mutation of VHL (von Hippel-Lindau) promote carcinogenesis, angiogenesis, and metabolic remodeling primarily through the HIF-related pathway, making it recognized as a key driver in both inherited VHL syndrome and sporadic ccRCC (4). Beyond age and gender, lifestyle-related factors such as obesity, hypertension, and smoking are well-established as being highly correlated with the occurrence of ccRCC (5). Typical clinical manifestations of flank pain, abdominal mass, and hematuria appear to be uncommon in newly diagnosed renal cell carcinoma (RCC) due to its location in the retroperitoneal space, and some patients are also accompanied by paraneoplastic syndrome (5). Most ccRCC patients are detected at an early stage, and those with localized ccRCC may achieve a cure through partial or radical nephrectomy (5). Small-molecule inhibitors targeting the activated VEGF, PDGF, and mTOR pathways, as well as single-agent or combination immunotherapy, serve as effective treatment for advanced or metastatic ccRCC (6). Nevertheless, clinical efficacy of these treatments is restricted by adverse responses and congenital and acquired drug resistance (6). Therefore, the exploration of new prognostic genes and potential mechanisms will not only help to understand the pathogenesis and molecular mechanisms of diseases but also offer targets for clinical diagnosis and treatment.

The ongoing improvements of RNA sequencing technology enable the analysis of transcriptome-wide gene expression variation with unprecedented accuracy and breadth. Single-cell RNA sequencing (scRNA-seq) is a cutting-edge technique for accurately quantifying the transcriptome characterization of individual cells (7). By measuring gene expression in different cells or at different time points, scRNA-Seq can assist in analyzing tumor heterogeneity, distinguish new cell subtypes, and identify cell growth and differentiation trajectories, providing powerful tools to investigate pathogenesis, biomarkers, drug resistance mechanisms, and new therapeutic targets in cancer (7). However, scRNA-seq fails to preserve the original location information of each cell, which is destroyed by the dissociation steps. Spatial transcriptomics RNA sequencing (stRNA-seq) addresses this limitation by capturing the spatial localization of RNA expression data in tissue slices using various technologies (8). The application of stRNA-seq is restricted by factors such as low spatial resolution, small sample size, and high cost (8). Integration of scRNA-seq data and spatial transcriptome (ST) data has the advantage of revealing spatial heterogeneity in different cell subtypes, thereby enhancing our understanding of the comprehensive molecular profile in the tumor microenvironment (9).

This study integrates scRNA-seq and ST data to systematically reveal, for the first time at single-cell resolution, the spatial distribution characteristics of cellular subpopulations within ccRCC tumors and their microenvironment. Unlike traditional bulk RNA sequencing-based prognostic models, our research focuses on cell types directly associated with disease progression, thereby overcoming limitations posed by cellular heterogeneity. We constructed a risk prediction model incorporating key genes, establishing an innovative molecular classification system for ccRCC that enables more accurate prognostic assessment. Furthermore, our study uncovers the dual role of key genes in both suppressing tumor angiogenesis and promoting M1 macrophage polarization, providing novel insights into microenvironmental interaction mechanisms. In conclusion, this research establishes a theoretical foundation for personalized treatment and novel targeted strategies in ccRCC.

## 2 Materials and methods

### 2.1 Data extraction

The University of California, Santa Cruz (UCSC) Xena database (https://xenabrowser.net/datapages/, GDC Data Release v18.0) was adopted to download the RNA-seq data and clinical information (age, gender, histological, stage, T stage, N stage, and M stage) of The Cancer Genome Atlas (TCGA)-ccRCC dataset, which was employed as a training set. This dataset contained tissue samples from 531 ccRCC patients with complete survival information and 72 normal control samples. The normalized TPM expression matrix was utilized for all analyses, except for the differential expression analysis where the Counts expression matrix was employed. To convert Ensemble IDs in the gene expression matrix into gene symbols, they were combined with the corresponding annotation files of the species. The International Cancer Genome Consortium (ICGC) database (https://dcc.icgc.org/) was employed to obtain the ICGC-ccRCC dataset (validation set), including tissue samples from 91 ccRCC patients with complete survival data and gene expression data. Additionally, the Gene Expression Omnibus (GEO) database (http://www.ncbi.nlm.nih.gov/geo/) was utilized for gaining the scRNA-seq dataset (GSE156632) and ST data (GSE175540). The GSE156632 dataset was acquired based on the sequencing platform GPL24676, comprising seven ccRCC tissue samples and five paraneoplastic tissue samples. Meanwhile, the GSE175540 dataset was obtained utilizing the sequencing platform GPL24676, encompassing 12 frozen ccRCC primary tumor samples and 12 formalin-fixed paraffin-embedded (FFPE) sectioned ccRCC primary tumor samples. All the above data were downloaded on April 15, 2024.

### 2.2 scRNA-seq data analysis

The GSE156632 dataset was utilized to investigate gene expression at the cellular level. Initially, scRNA-seq data were generated as Seurat objects using the Seurat package (v 5.0.1) (10). Subsequent quality control (QC) was performed to ensure the acquisition of high-quality cells. To this end, the number of genes detected per cell was set between 200 and 10,000 to prevent the inclusion of empty droplets or low-quality cells and to avoid multiple cells being incorrectly identified as one (11, 12). Additionally, the mitochondrial gene percentage was limited to below 10% to exclude stressed or apoptotic cells and reduce background noise (11, 12). Together, these criteria ensured the reliability of the cells used in subsequent analyses. Following QC, a global scaling normalization method (LogNormalize) was employed to normalize the feature expression measurements of each cell according to the total expression, followed by multiplication with a scaling factor (default 10,000) and logarithmic transformation of the results. The Find Variable Features function was applied to identify the highly variable genes. After that, linear dimensionality reduction was performed to determine the optimal linear dimensionality value for cell clustering based on scree plot analysis. Furthermore, cells were clustered according to this optimal linear dimensionality value (resolution = 0.1). Additionally, batch effects between groups were assessed and removed if present. Importantly, cell clusters obtained from clustering were annotated based on marker genes derived from literature sources (13). Finally, the distribution of these cell types between ccRCC and control groups was presented as percentages (%).

### 2.3 ST data analysis

In the ST data analysis, we first imported the raw expression matrix using the Load10X_Spatial function from the Seurat package and performed quality control for spots in each sample. We calculated the number of detected genes per spot (nFeature_spatial), the total UMI count (nCount_spatial), and the mitochondrial gene percentage (percent.mt). To exclude empty or low-quality spots, we filtered out those with fewer than 200 detected genes or a mitochondrial gene ratio exceeding 10%. Subsequently, the SCTransform method was applied to normalize the quality-controlled data and select highly variable genes (variable.features. n = 3,000), thereby reducing technical noise and mitigating the influence of sequencing depth. Based on the normalized expression matrix, batch effect correction and data integration were performed using the FindIntegrationAnchors and IntegrateData functions. The integrated data were then subjected to dimensionality reduction via principal component analysis (PCA), followed by clustering analysis and Uniform Manifold Approximation and Projection (UMAP) visualization.

We further visualized the spatial distribution of nFeature and nCount in each sample of the GSE175540 dataset using SpatialFeaturePlot. Next, the FindAllMarkers function was employed to identify marker gene sets (set 1 and set 2) across different cell types in the GSE156632 and GSE175540 datasets, respectively. The thresholds were set as “avg_log2Fold Change (FC) > 0.3 & Padj< 0.05 & d > 0.2” for set 1 and “avg_log_2_FC > 0.3 & Padj< 0.05 & d > 0.1” for set 2, and duplicate genes were removed from the identified markers. Finally, multiple imputation analysis (MIA) was conducted based on a hypergeometric test to evaluate the enrichment levels of these two marker gene sets across different spatial regions. This allowed identification of the predominant cell types in each region, and highly enriched cell types were selected and recorded as target cells for subsequent analysis.

### 2.4 Functional analysis of target cells

To investigate the heterogeneity of target cells, the expression profile of these cells was first extracted from the GSE156632 dataset, and then the secondary clustering analysis was performed on them. The resulting cell subsets were annotated and visualized using UMAP for better understanding (resolution = 0.1). Furthermore, a bubble plot was generated to visualize the expression patterns of marker genes among different subgroups. Functional enrichment analysis of target cells was implemented to explore their signaling pathways associated with ccRCC progression using the ReactomeGSA package (v 1.12.0) (*P* < 0.05) (14). Based on the CellChatDB.human as a reference, the CellChat package (v 1.6.1) (15) was employed to perform cell communication analysis on the target cells and other cells annotated to the GSE156632 dataset. Additionally, the Monocle 2 package (v 2.22.0) (16) was utilized to conduct a pseudotime analysis of the target cells, aiming to elucidate their differentiation trajectory, followed by a comparison of the different subtypes of this target cell’s differentiation trajectories between ccRCC and control groups.

### 2.5 Recognition of candidate genes

In the GSE156632 dataset, we used the FindMarkers function from the Seurat package to identify differentially expressed genes 1 (DEGs1) associated with target cells between the ccRCC and control groups, with filtering thresholds set at |avg_log_2_FC| ≥ 0.25 and P< 0.05. The resulting gene set was deduplicated, and DEGs1 were subsequently visualized using a Manhattan plot generated with the scRNAtoolVis package (v0.0.7). For the TCGA-ccRCC dataset, differential expression analysis was performed using the DESeq2 package (v1.38.0), which models raw gene counts using a negative binomial distribution, normalizes data by estimating size factors and dispersion parameters, and assesses the significance of expression differences via the Wald test. The Benjamini–Hochberg method was applied to control the false discovery rate (FDR). Genes meeting the criteria of FDR< 0.05 and |log_2_FC| > 2 were defined as differentially expressed genes 2 (DEGs2). Furthermore, a volcano plot was generated using the ggplot2 package (v3.4.4) to visualize the distribution of DEGs2, in which the top 10 upregulated and downregulated genes, ranked by |log_2_FC|, were labeled. A heatmap illustrating the expression patterns of these 20 key DEGs2 was also created using the pheatmap package (v1.0.12). Finally, candidate genes for subsequent analysis were obtained by taking the intersection of DEGs1 and DEGs2.

### 2.6 Identification of prognostic genes

In the TCGA-ccRCC dataset, candidate genes were subjected to univariate Cox regression analysis using the survival package (v 3.5-3) (17) with hazard ratio (HR) ≠ 1 & P< 0.0001 as the screening condition, thereby identifying genes significantly associated with ccRCC prognosis. Subsequently, these identified genes underwent the proportional hazards (PH) hypothesis tests (*P* > 0.05), and only those passing this test were further included in least absolute shrinkage and selection operator (LASSO) regression analysis employing the glmnet package (v 4.1.4) (18) for additional screening. Furthermore, a stepwise regression analysis (direction = both) was performed on the genes obtained from LASSO analysis to select prognostic genes for generating the risk model in ccRCC samples.

### 2.7 Construction and validation of the risk model

Based on 531 ccRCC samples with complete survival data in the TCGA-ccRCC dataset, a risk model was created using the prognostic genes identified in the above analysis. The risk score formula was as follows: risk score = $\sum_{\text{i}=1}^{\text{n}}\left({\text{β}}_{\text{i}}\times {\text{X}}_{\text{i}}\right)$. Among them, βi and Xi represented the coefficient obtained by stepwise regression analysis and relative expression of prognostic gene i, respectively. Then, among the 531 ccRCC samples in the TCGA-ccRCC dataset, a risk score was calculated for each ccRCC patient based on the risk score calculation formula. The ccRCC samples were categorized into high and low risk cohorts based on the best cutoff value of risk score. The risk curve and survival status map were drawn to reveal the distribution of samples, followed by heat maps illustrating the expression patterns of prognostic genes across the two risk cohorts. Furthermore, the survminer package (v 0.4.9) (19) was employed to conduct Kaplan–Meier (K-M) survival analysis for comparing survival differences between the two risk cohorts (*P* < 0.05). At the same time, the receiver operating characteristic (ROC) curve analysis was implemented using the survival ROC package (v 1.0.3) (20), followed by the calculation of the area under the curve (AUC) values for risk model at 1, 3, and 5 years to assess its effectiveness. To validate the generalizability of the model, we performed external validation using the independent ICGC-ccRCC dataset. First, standardized preprocessing—including TPM conversion and gene symbol harmonization—was applied to the validation set expression matrix to maintain consistency with the training set, and the same feature genes were extracted. Subsequently, the gene coefficients derived from the training set were used to calculate risk scores for each validation sample. Patients were then stratified into high- and low-risk groups based on the optimal cutoff value. Finally, survival analysis and evaluation of model performance metrics were conducted to verify the robustness of the risk model.

### 2.8 Generation and evaluation of nomogram

In the TCGA-ccRCC dataset, risk score and other clinical indicators (risk score, age, gender, histological, stage, T stage, N stage, and M stage) were integrated, and then they were sequentially subjected to univariate Cox regression analyses (*P* < 0.05), PH hypothesis test (*P* > 0.05), and multivariate Cox regression analyses (*P* < 0.05), aiming to obtain independent prognostic factors. Subsequently, a nomogram was constructed based on these identified independent prognostic factors using the rms package (v 6.5-0) (21). In the nomogram, each independent prognostic factor was assigned a corresponding score, and the sum of these scores determined the total points. Following this, the 1-, 3-, and 5-year survival rates of ccRCC patients were predicted according to this total points. Furthermore, the predictive ability of the nomogram model was assessed by calibration curve and decision curve. Specially, the decision curve was realized by the ggDCA package (v 1.2) (https://rdrr.io/github/yikeshu0611/ggDCA/).

### 2.9 Enrichment analysis between high risk and low risk cohorts

Gene set enrichment analysis (GSEA) was implemented to understand the biological functions and pathways enriched in ccRCC patients from high- and low-risk cohorts. Differential expression analysis was performed on the two risk cohorts using the DESeq2 package to obtain the corresponding genes and their corresponding log2FC values, and the obtained genes were sorted in descending order based on the log2FCs. Next, the “c2.cp.kegg.v7.5.1.symbols.gmt” gene set in the Molecular Signatures Database (MSigDB) (https://www.gsea-msigdb.org/gsea/msigdb/index.jsp) was used as the reference gene set, and the clusterProfiler package (v 4.8.2) (22) was utilized to perform GSEA (*P* < 0.05). In addition, the significantly enriched pathways were visualized using the enrichplot package (v 1.18.3) (23). Gene set variation analysis (GSVA) was also conducted to explore the difference in enrichment pathways between the two risk cohorts, with the “h.all.v2022.1.Hs.symbols.gmt” gene set employed as the background gene set (*P* < 0.05).

### 2.10 Immunocyte infiltration analysis

To explore the immune infiltration status across all samples, the CIBERSORT algorithm was applied to assess the proportions of 22 different infiltrating immune cell types (24) in the two groups of the training set, and the “ggplot2” (v 3.4.4) was used to draw a stacked diagram. Subsequently, the infiltration differences of 22 immune cells between the two risk cohorts in the training set were compared by the Wilcoxon test (*P* < 0.05), and the immune cells exhibiting significant differences were recorded as differential immune cells. Furthermore, the relationships between immune cells and prognostic genes in the training set were explored using the correlation analysis method of Mantle test (*P* < 0.05).

### 2.11 Drug sensitivity analysis

To further explore the potential guidance of risk scores for chemotherapy, the half-maximal drug inhibitory concentration (IC50) values for 138 drugs were obtained using the oncopdict package (v 0.2) (25) in the Genomics of Drug Sensitivity in Cancer (GDSC) (Version number: Release 8.5) database (https://www.cancerrxgene.org). Then, Spearman correlation analysis was implemented using the psych package (v 2.2.9) (26) to explore the correlation between the IC50 values of drugs and risk scores in the TCGA-ccRCC dataset (|correlation coefficient (cor)| > 0.3 & *P* < 0.05) (27). Furthermore, the Wilcoxon test was adopted to compare differences in IC_50_ values of drugs screened by Spearman correlation analysis between the two risk cohorts (*P* < 0.05).

### 2.12 Prognostic gene expression assessment

Prognostic gene expression was assessed. First, the expression of prognostic genes in target cells was evaluated and demonstrated by UMAP plots. Second, its expression in the cells annotated by the scRNA-seq data analysis was evaluated and represented by violin plots. Furthermore, the expression of prognostic genes in each sample of the ST dataset (GSE175540) was explored. In addition, based on the common immune cells (T cells, CD8 T cells, Natural Killer (NK) cells, B lineage, monocytic lineage, neutrophils, endothelial cells, and fibroblasts) from the published literature (28), we explored the expression of prognostic genes in these immune cells.

### 2.13 Cell culture and transfection

HK-2, ACHN, 786-O, HUVEC, and THP-1 were purchased from the American Type Culture Collection (ATCC, Manassas, VA, USA). These cells were cultured with RPMI-1640 medium (Gibco, Grand Island, NY, USA) under 5% CO_2_ atmosphere to maintain cellular homeostasis. The medium was enriched with 10% fetal bovine serum (FBS, Thermo Fisher Scientific, Waltham, MA, USA), along with 100 µg/mL penicillin and 100 μg/mL streptomycin (Sigma-Aldrich, St. Louis, MO, USA). Phorbol-12-myristate-13-acetate (10 ng/mL, PMA, Sigma, St. Louis, MO, USA) was used to induce THP-1 differentiation for 24 h. Mycoplasma contamination detection was performed using a mycoplasma detection kit (Lonza, Basel, Switzerland) to ensure cell integrity. To obtain the nucleotide sequence of the ATP1A1, the sequence was comprehensively retrieved from the National Center for Biotechnology Information (NCBI) database. ATP1A1’s coding sequence was amplified and subsequently introduced into the PcDNA3.0 (GenePharma, Shanghai, China), and the lentiviral vector (oe-ATP1A1) and control vector (oe-NC) were constructed. Transfection of the plasmid into 786-O cells was achieved by Lipofectamine 3000 reagent (Invitrogen, Carlsbad, CA, USA).

### 2.14 RNA extraction, reverse transcription, and quantitative reverse transcription polymerase chain reaction

Total RNA was isolated from 786-O and THP-1 cultured cells-using TRIzol reagent (Invitrogen). Following isolation, reverse transcription was conducted with the PrimeScript RT Master Mix Kit (TaKaRa, Dalian, China), strictly following the kit instruction. The mRNA expression levels of ATP1A1, IL-6, TNF-α, CD80, CD206, CD163, and IL-10 were detected by SYBR Green fluorescence (G3326, Servicebio, Wuhan, China), and we computed the expression of the genes by means of the 2^−ΔΔCT^ method. Primer sequences are detailed in **Supplementary Table S1**. The expression of target gene was corrected with GAPDH as an internal control. PCR reaction conditions were 95°C 5 S, 63°C 30 S, 72°C 30 S for 35 cycles.

### 2.15 Transwell assay

For migration assay, human umbilical vein endothelial cells (HUVECs) were treated with different cell media (CM) from 786-O cells for 48 h and cells were resuspended in 200 μL of FBS-free medium (1 × 10^5^ cells) and added to the top chamber (BD Bioscience, Franklin Lakes, NJ, USA) of Transwell (8 μm wells). The lower chamber was added with the medium supplemented with 10% FBS. After 24 h, cells were fixed, stained, and counted under a microscope.

### 2.16 Tube formation assay

For the tube formation assay, HUVECs were treated with different CM from 786-O cells for 48 h and then seeded in Matrigel (354234, BD Bioscience, Franklin Lake, New Jersey, USA) for 6–8 h. Tube-like structures were imaged under a light microscope (Olympus, Tokyo, Japan). The number of nodes of cells was quantified via ImageJ software (version 1.8.0; National Institutes of Health).

### 2.17 Enzyme-linked immunosorbent assay

To assess the polarization state of macrophages, macrophages cocultured with conditioned media from different treatment group cancer cells were collected. The expression levels of CD80 and CD163 were then measured in cell lysates using specific enzyme-linked immunosorbent assay (ELISA) kits (Abcam, Cambridge, United Kingdom), following the manufacturer’s instructions (29–31).

### 2.18 Statistical analysis

R software (v 4.2.2) was adopted to process and analyze the data. Differences between the two groups were compared through the implementation of Wilcoxon test. Each experiment was performed at three times. The P value less than 0.05 was considered statistically significant.

## 3 Results

### 3.1 A total of five cell types were generated by annotation

**Figure 1:**
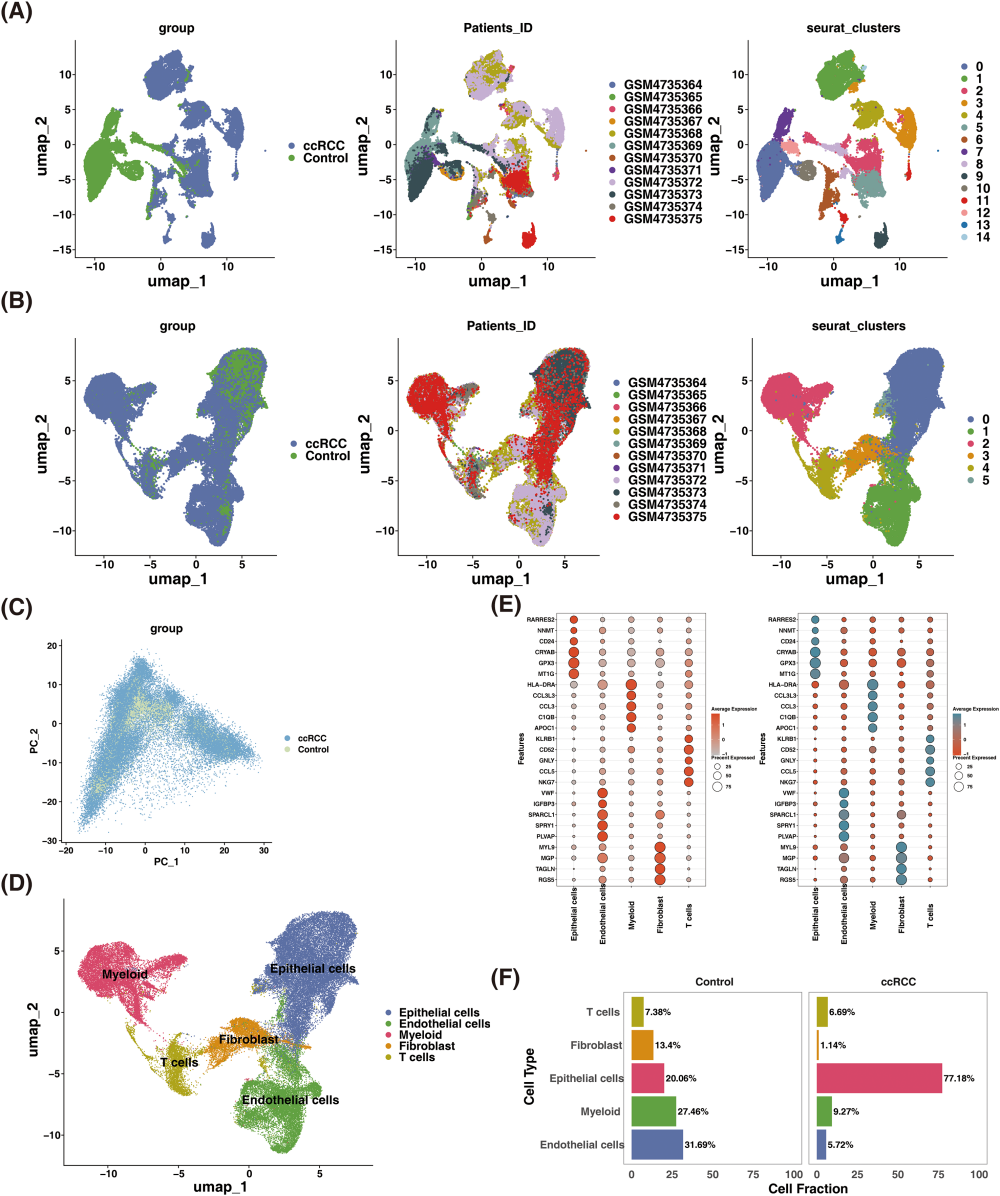
A total of five cell types were generated by annotation. **(A)** UMAP diagram before batch effect correction. **(B)** UMAP plot after batch effect correction. **(C)** PCA plot of the disease and control groups after batch effect correction. **(D)** UMAP clustering map annotated for five cell types. **(E)** Bubble plot of marker genes distinguishing different cell types. **(F)** Visualization plot of cell group proportion. UMAP, uniform manifold approximation and projection; PCA, principal component analysis.

Before QC, the GSE156632 dataset exhibited a concentration of gene detection below 7,500 in the cells, with a sum of expression levels below 50,000 for all genes (**Supplementary Figure 1A**). Meanwhile, mitochondrial genes were found to be concentrated below 15%. The dataset consisted of a total of 54,988 cells. After QC procedures, we obtained a refined dataset comprising 53,164 cells and 22,064 genes (**Supplementary Figure 1B**). Through the application of FindVariableFeatures function, 2,000 highly variable genes after QC were selected for further analysis (**Supplementary Figure 1C**). Subsequently, the optimal linear dimensionality value was determined to be 20 (**Supplementary Figure 1D**). Following this, based on this optimal linear dimensionality value, a cluster analysis yielded 15 cell clusters, revealing a significant batch effect between groups (**Figure 1A**). Upon removing this batch effect, six cell taxa were identified through cluster analysis (**Figure 1B**). Furthermore, the results of PCA demonstrated an absence of distinct boundaries among the groups (**Figure 1C**). Furthermore, five cell types were generated by annotation, containing fibroblasts (RGS5, TAGLN, MGP, MYL9), endothelial cells (PLVAP, SPRY1, SPARCL1, IGFBP3, VWF), T cells (NKG7, CCL5, GNLY, CD52, KLRB1), myeloid cells (APOC1, C1QB, CCL3, CCL3L3, HLA-DRA), and epithelial cells (MT1G, GPX3, CRYAB, CD24, NNMT, RARRES2) (**Figure 1D**). The expression of marker genes in these cells was demonstrated by bubble plots (**Figure 1E**). Additionally, the percentages of these five cell types between the ccRCC and control groups were compared, revealing that epithelial cells exhibited the highest prevalence in the ccRCC group and were significantly more abundant compared with the control group (**Figure 1F**).

### 3.2 Epithelial cells were identified as target cells

**Figure 2:**
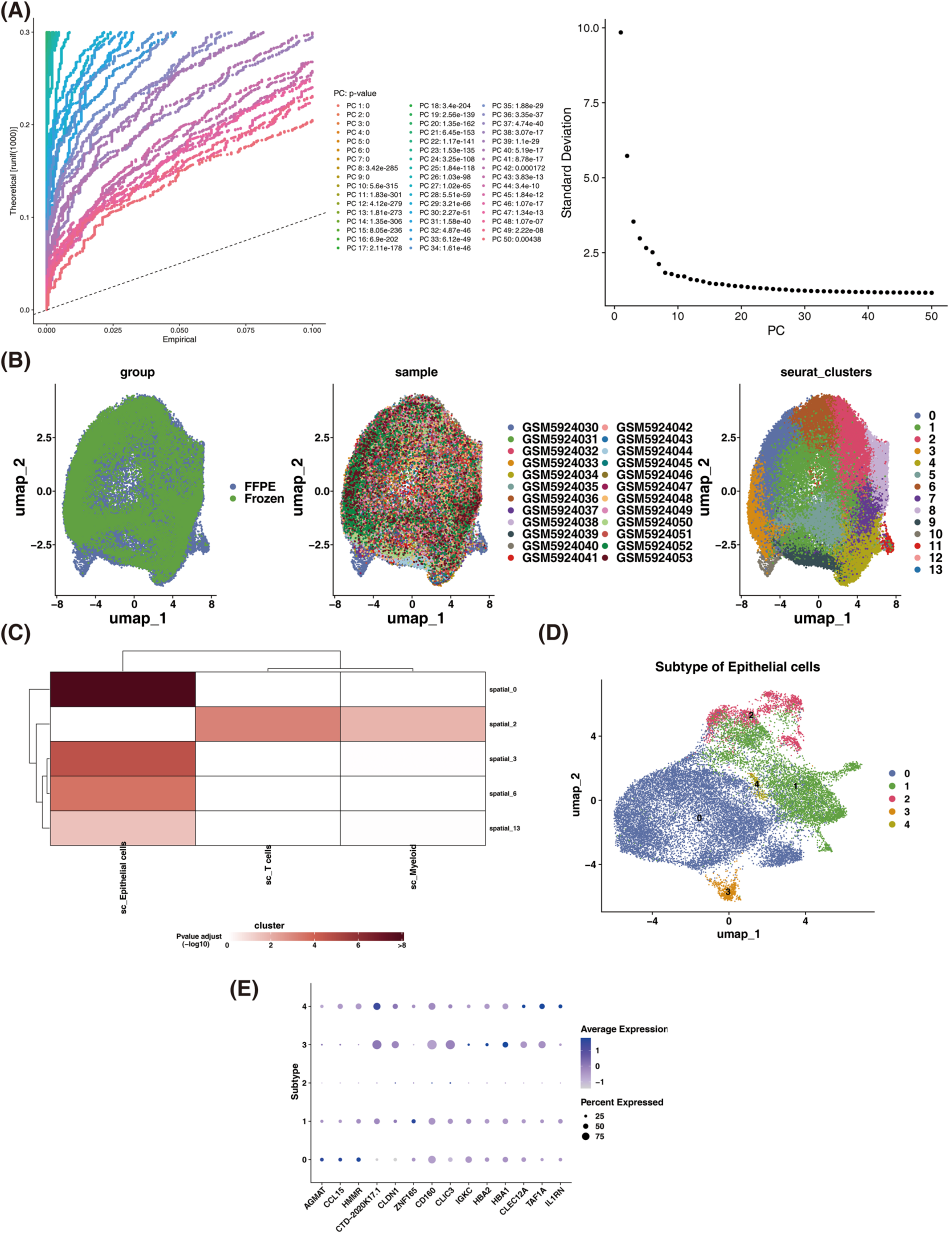
Epithelial cells were identified as target cells. **(A)** Plot of results for PCA-based dimensionality reduction analysis. **(B)** UMAP plot of ST data after batch effect correction. **(C)** MIA analysis of characterization genes. **(D)** Heterogeneity analysis of epithelial cells. **(E)** Visualization of expression between marker gene subgroups. UMAP, uniform manifold approximation and projection; PCA, principal component analysis; ST, spatial transcriptomics; MIA, multiple imputation analysis.

In the GSE175540 dataset, the nFeature and nCount for each sample were presented in Attachment **Supplementary Figure 2** and **Supplementary Figure 3**. After normalization, the linear dimensionality value for the GSE175540 dataset was determined to be 20 (**Figure 2A**). Subsequently, cell clusters were classified based on this value as well as batch effect correction between multiple samples. After downscaling cluster analysis, the cells in the GSE175540 dataset could be categorized into 14 cellular subpopulations (**Figure 2B**). In addition, the spatial distribution of subpopulations in each sample in this dataset was visualized using the SpatialFeaturePlot function (**Supplementary Figure 4**). After that, based on 476 feature genes1 and 622 feature genes2 identified in the GSE156632 and GSE175540 datasets, respectively, MIA was implemented to identify target cells. The results revealed that epithelial cells were the most highly enriched and were recorded as target cells for subsequent analysis (**Figure 2C**). Furthermore, epithelial cells were clustered into five subpopulations (**Figure 2D**), followed by bubble plots showing the expression of marker genes between subpopulations (**Figure 2E**).

### 3.3 Functions of epithelial cells were characterized

**Figure 3:**
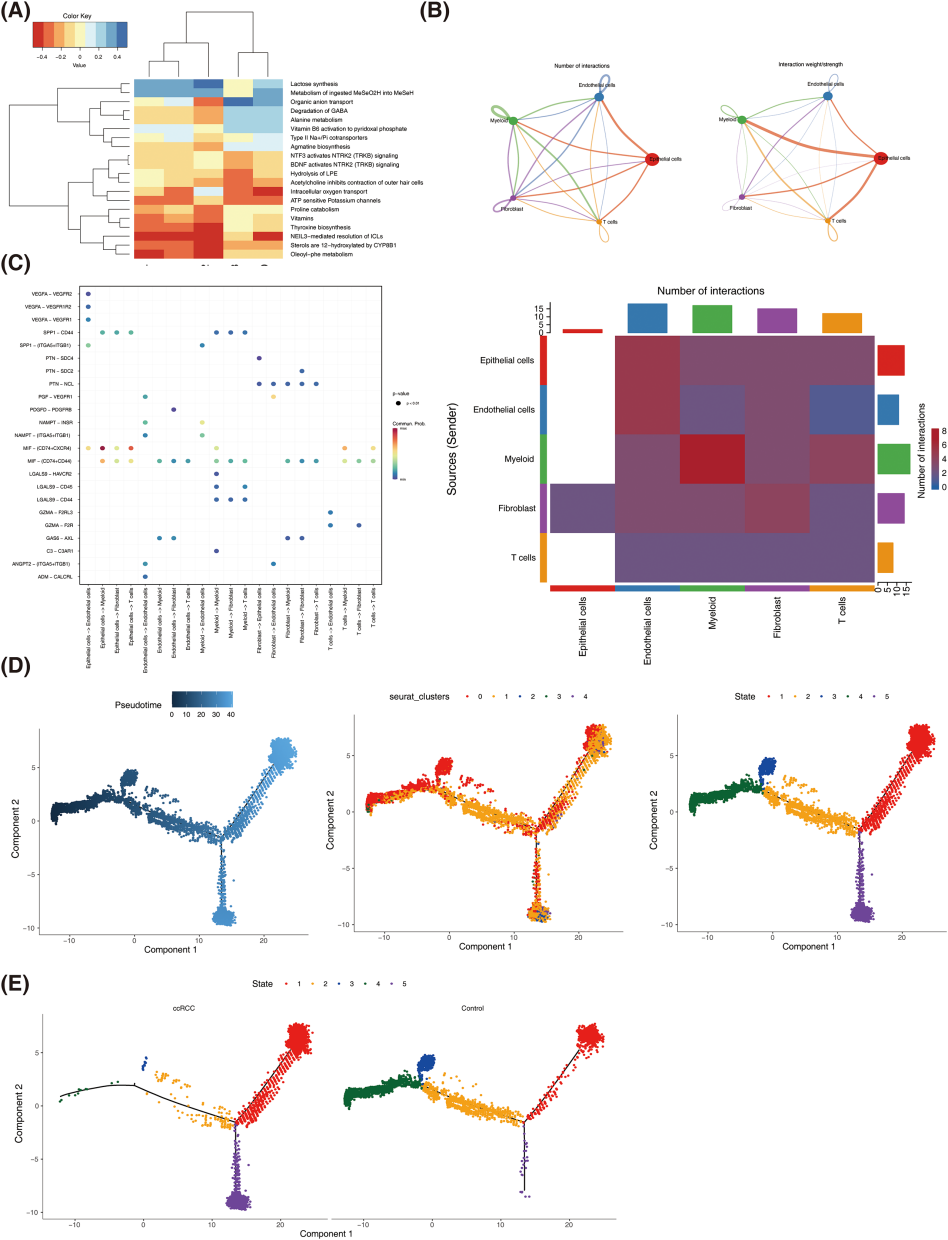
Functions of epithelial cells were characterized. **(A)** Heatmap of functional enrichment analysis of target epithelial cells. **(B)** Cell–cell communication interaction plot between endothelial cells and four other cell types. **(C)** Plot of cellular communication receptor ligands (left), and diagram of interactions and quantities (right). **(D)** Diagram of pseudotime analysis trajectory: (D-1) trajectory plots of different cell subpopulations (D-2) and stage map of different differentiation trajectories (D-3). **(E)** Differentiation stage map of different differentiation trajectories between normal and disease groups.

With the application of the ReactomeGSA package, 1,766 signaling pathways significantly enriched by epithelial cells were identified. Among them, the top 20 signaling pathways were selected to show, including “organic anion transport”, “degradation of GABA”, “alanine metabolism”, “proline catabolism”, etc. (**Figure 3A**). Then, cell communication analysis revealed that there were multiple communication relationships between endothelial cells and the other four kinds of cells, and the relationship was strong (**Figure 3B**). Meanwhile, the interaction between epithelial cells and myeloid was mediated cells by migration inhibitory factor (MIF)-(CD74+CXCR4) (**Figure 3C**). Furthermore, the pseudotime analysis was adopted to assess the differentiation status of epithelial cells. The results demonstrated that epithelial cells were differentiated as differentiating from left to right over time (**Figure 3D**) and from normal to disease (**Figure 3E**). These results suggested that epithelial cells played a moderately important role in the genesis and development of ccRCC.

### 3.4 A sum of seven prognostic genes model was recognized for ccRCC

**Figure 4:**
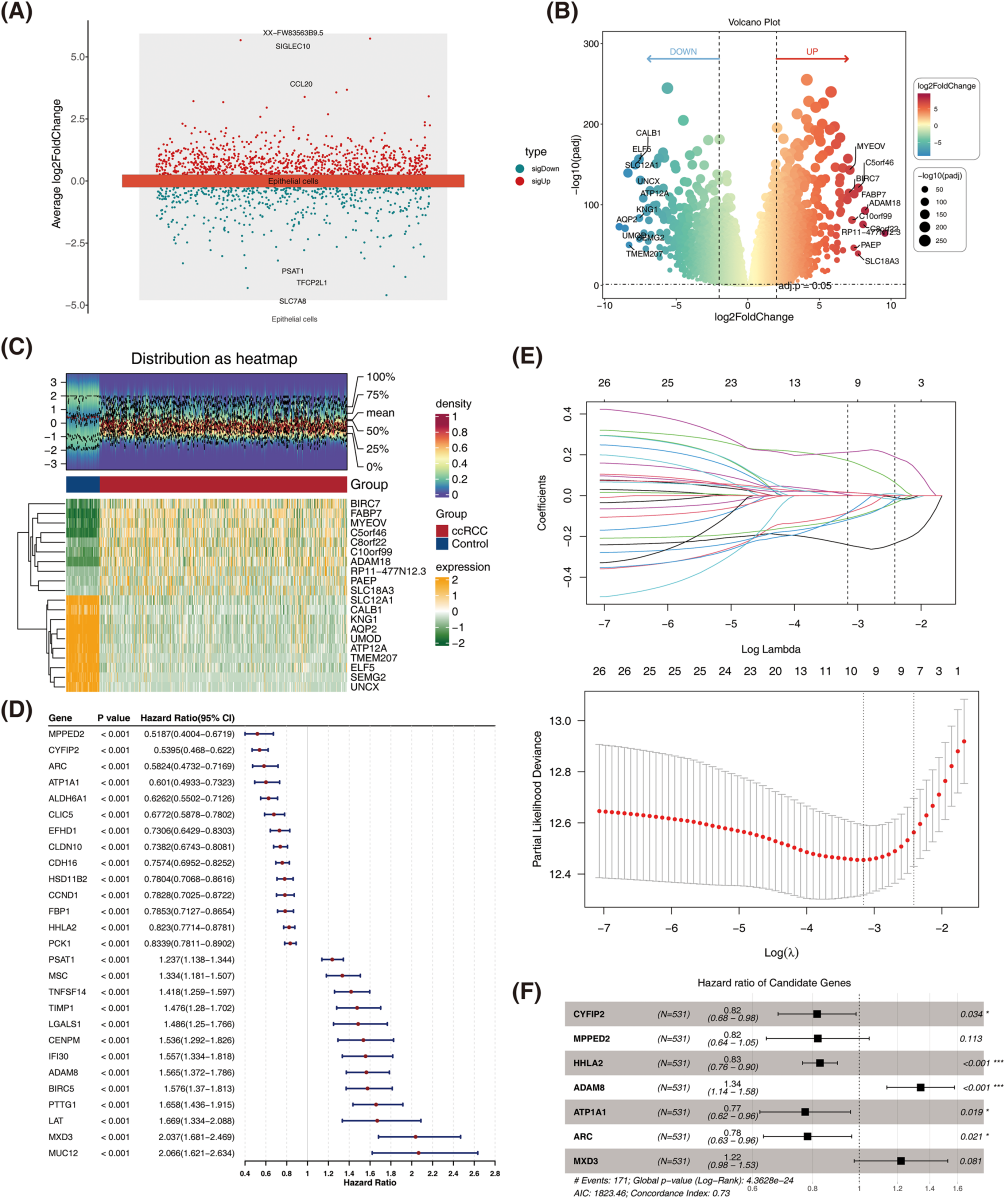
A sum of seven prognostic genes were recognized for ccRCC. **(A)** Manhattan plot for identification of differentially expressed genes. **(B)** Volcano plot of differentially expressed genes. Red color indicates up-regulation of gene expression, and green color indicates down-regulation of gene expression. **(C)** Heatmap of differentially expressed genes. Orange represents high expression, and green represents low expression. **(D)** Forest plot of univariate Cox proportional hazards regression for candidate genes. **(E)** LASSO regression for further screening of prognostic genes. **(F)** Results of stepwise regression analysis. LASSO, least absolute shrinkage and selection operator; ccRCC, clear cell renal cell carcinoma. *P < 0.1 and ***P < 0.001.

After differential analysis, 1,305 DEGs1 (834 up-regulated genes and 471 down-regulated genes) associated with epithelial cells were identified between ccRCC and control groups in the GSE156632 dataset (**Figure 4A**). Meanwhile, 2,323 DEGs2 were identified between ccRCC and control groups in the TCGA-ccRCC dataset, containing 1,514 up-regulated genes and 809 down-regulated genes (**Figures 4B, C**). Subsequently, 226 candidate genes were obtained by overlapping 1,305 DEGs1 and 2,323 DEGs2. Afterward, these 226 candidate genes were subjected to univariate Cox regression analysis, resulting in the identification of 27 genes significantly associated with the prognosis of ccRCC (HR ≠ 1 & *P* < 0.0001) (**Figure 4D**). These 27 genes were passed in the PH hypothesis test (*P* > 0.05). Furthermore, 10 genes were selected by LASSO analysis, namely, CYFIP2, MPPED2, TIMP1, HHLA2, TNFSF14, PSAT1, ADAM8, ATP1A1, ARC, and MXD3 (lambda = 0.04244543) (**Figure 4E**). Finally, through the implementation of stepwise regression analysis, CYFIP2, MPPED2, HHLA2, ADAM8, ATP1A1, ARC, and MXD3 were selected and included in the subsequent analysis as prognostic genes (**Figure 4F**).

### 3.5 Risk model was effective in predicting prognosis of ccRCC

**Figure 5:**
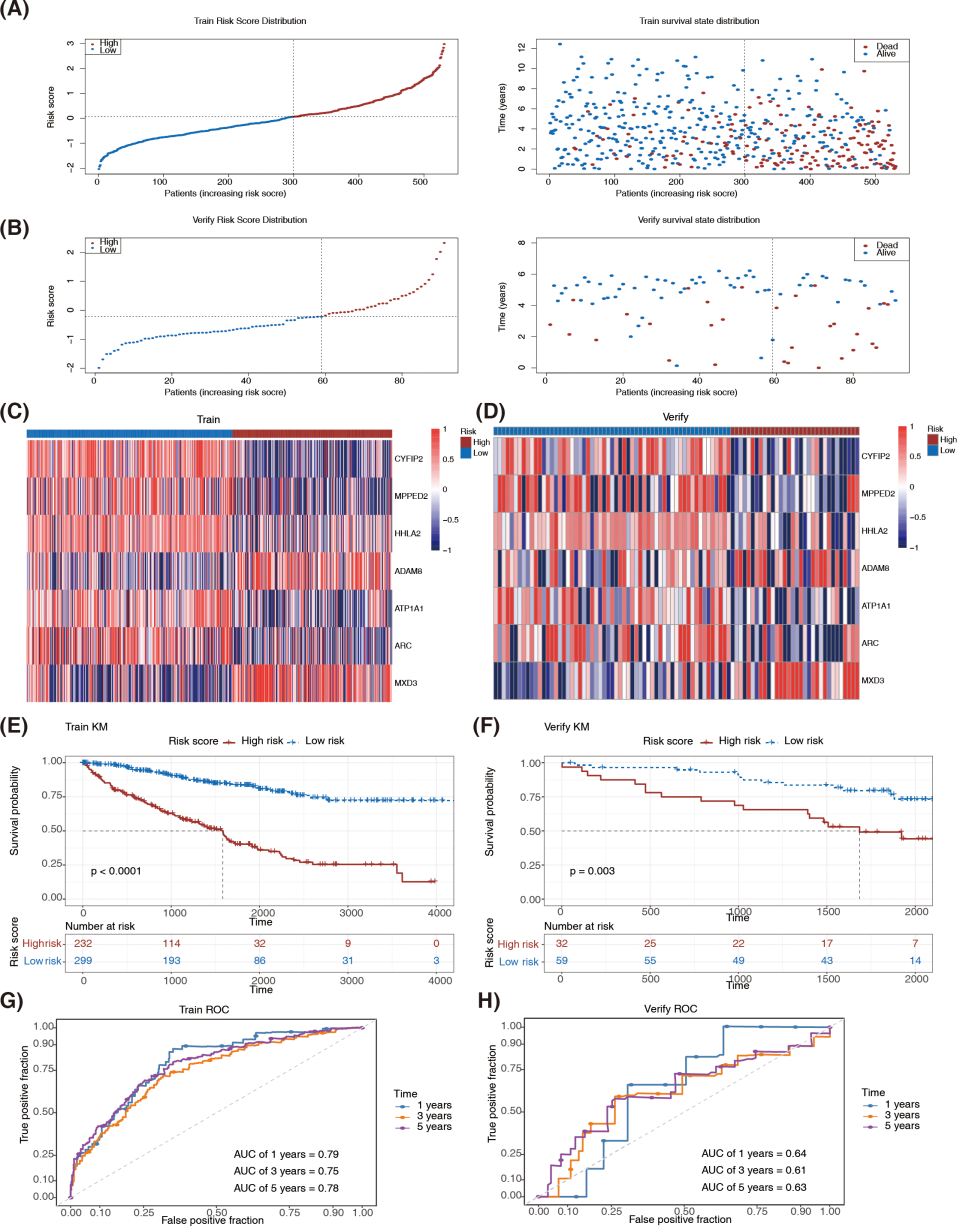
Risk model was effective in predicting prognosis of ccRCC. **(A)** Risk curve and survival status scatter plots in TCGA-ccRCC dataset. **(B)** Risk curve and survival status plots in the ICGC-ccRCC dataset. **(C)** Heatmap of prognostic genes’ expression in TCGA-ccRCC dataset. High expression in red, low expression in blue. **(D)** Heatmap of prognostic genes’ expression in the ICGC-ccRCC dataset. High expression in red, low expression in blue. **(E)** K-M curve for high- and low-risk groups in the TCGA-ccRCC dataset. **(F)** K-M curve for high- and low-risk groups in the ICGC-ccRCC dataset. **(G)** ROC curve for high- and low-risk groups in TCGA-ccRCC dataset. **(H)** ROC curve for high- and low-risk groups in the ICGC-ccRCC dataset. TCGA, the cancer genome atlas; ccRCC, clear cell renal cell carcinoma; ICGC, international cancer genome consortium; KM, Kaplan–Meier; ROC, receiver operating characteristic.

Based on the seven prognostic genes identified in the above stepwise regression analysis, a risk model was constructed in the TCGA-ccRCC dataset. The risk score was calculated as follows: risk score = CYFIP2 expression × (−0.2019) + MPPED2 expression × (−0.1985) + HHLA2 expression × (−0.1881) + ADAM8 expression × 0.2933 + ATP1A1 expression × (−0.2590) + ARC expression × (−0.2473) + MXD3 expression × 0.1993. The ccRCC patients were divided into high-risk cohort (n = 232) and low-risk cohort (n = 299) according to the best cutoff value (0.0617) of the risk score. Meanwhile, the ccRCC patients in the ICGC-ccRCC datasets were also divided into high-risk cohort (n = 32) and low-risk cohort (n = 59) according to the best cutoff value (−0.2214) of the risk score. The risk curve and survival status map demonstrated that in both TCGA-ccRCC (**Figure 5A**) and ICGC-ccRCC datasets (**Figure 5B**), as the risk score increased, the number of deaths increased. The heat map indicated that prognostic genes showed consistent expression trends in both datasets. Specifically, ADAM8 and MXD3 were expressed at higher levels in the high-risk cohort, whereas CYFIP2, MPPED2, HHLA2, ATP1A1, and ARC were expressed at higher levels in the low-risk cohort (**Figures 5C, D**). Moreover, the K-M survival analysis indicated a marked difference in patient survival between the two risk cohorts in both TCGA-ccRCC (*P* < 0.0001) (**Figure 5E**) and ICGC-ccRCC datasets (*P* = 0.003) (**Figure 5F**), with a worse survival rate for all patients in the high-risk cohort. In addition, in the TCGA-ccRCC dataset, the AUC values at 1, 3, and 5 years were 0.79, 0.75, and 0.78, respectively (**Figure 5G**), whereas in the ICGC-ccRCC dataset, the AUC values at 1, 3, and 5 years were 0.64, 0.61, and 0.63, respectively (**Figure 5H**), revealing that the risk model performed better in predicting the ccRCC prognosis.

### 3.6 An effective nomogram was generated in ccRCC

**Figure 6:**
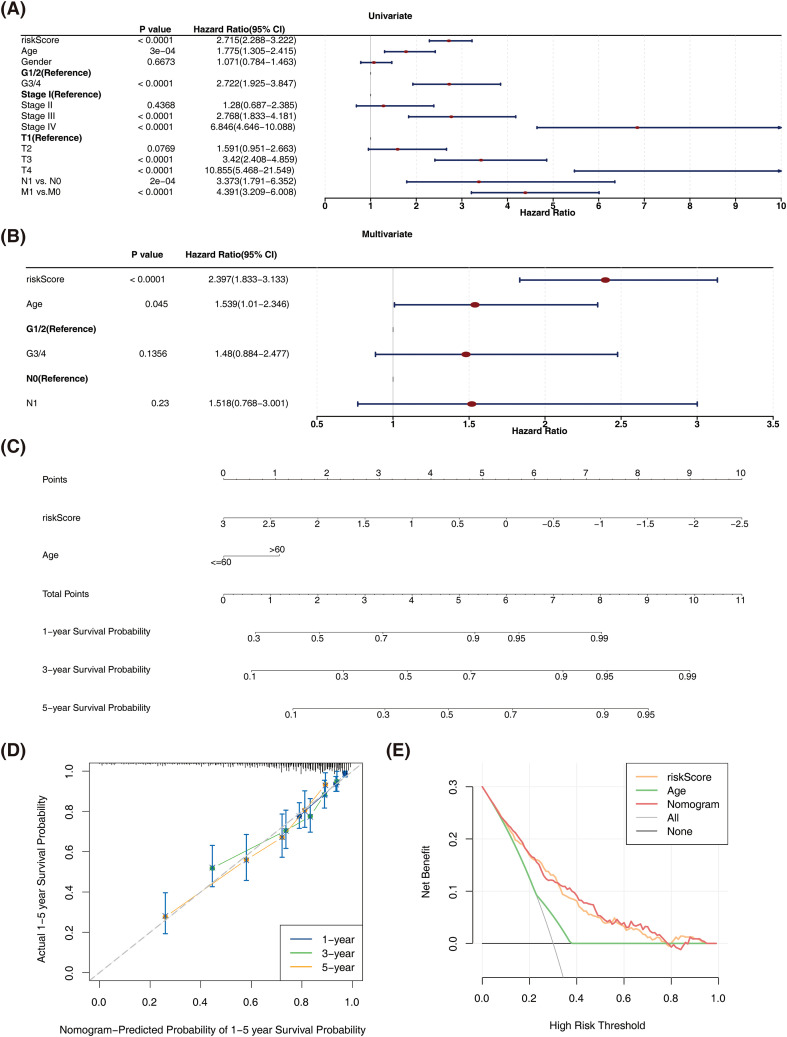
An effective nomogram was generated in ccRCC. **(A)** Forest plot for univariate Cox proportional hazards regression clinical indicators. **(B)** Forest plot of multivariate Cox regression clinical indicators. **(C)** Nomogram predicting 1-, 3-, and 5-year survival in patients with ccRCC. **(D)** Calibration curve for nomogram. **(E)** Decision curve analysis for nomogram. ccRCC, clear cell renal cell carcinoma; DCA, decision curve analysis.

Risk score and age were identified as independent prognostic factors through sequential screening by univariate Cox regression analysis (*P* < 0.05) (**Figure 6A**), PH hypothesis test (*P* > 0.05), and multivariate Cox regression analysis (*P* < 0.05) (**Figure 6B**). Following this, a nomogram was generated to forecast 1-, 3-, and 5-year survival of ccRCC patients (**Figure 6C**). Furthermore, the calibration curve of the nomogram demonstrated a high level of concordance with the reference line in terms of predicting 1-, 3-, and 5-year survival probabilities (**Figure 6D**). Additionally, the decision curve analysis (DCA) revealed that the overall predictive performance of the nomogram surpassed that of individual variables (**Figure 6E**), thereby indicating its superior accuracy in prognostication.

### 3.7 The relationships between the immune cells and the ATP1A1 were identified

**Figure 7:**
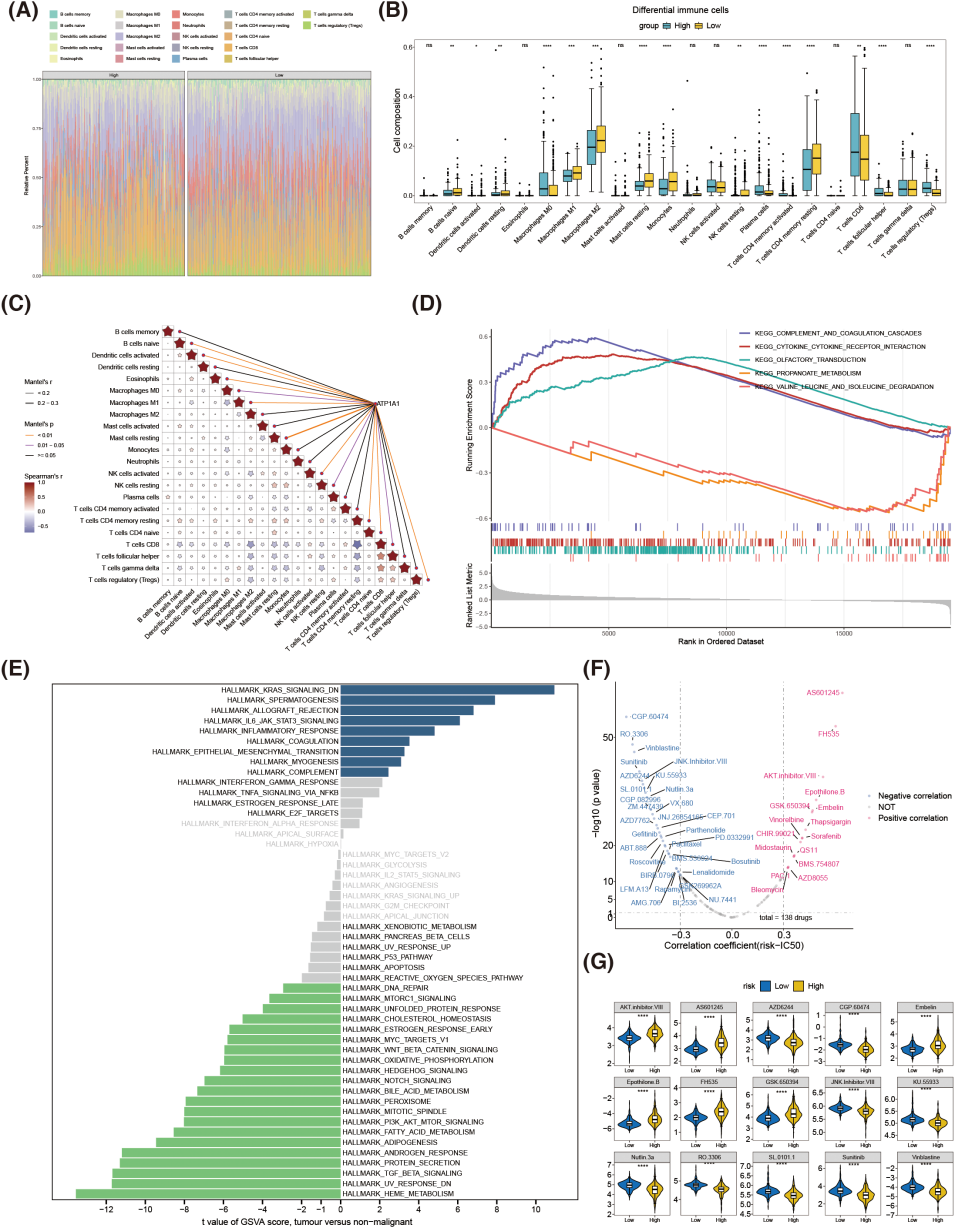
Multi-omics analysis of the risk groups: immunocyte infiltration, pathway activity, and drug sensitivity. **(A)** Composition of immune cell infiltration in high- and low-risk groups. **(B)** Box plots comparing the abundance of immune cell types that were significantly different between the high- and low-risk groups. **(C)** Correlation between differential immune cells and ATP1A1. **(D)** GSEA highlighting biological pathways enriched in the high-risk group compared with the low-risk group. **(E)** Results of GSVA for high- and low-risk groups. **(F)** Correlation analysis between drug IC_50_ values and risk scores. **(G)** Differences in the values of IC_50_ for common chemotherapeutic agents between high- and low-risk groups. GSEA, gene set enrichment analysis; GSVA, gene set variation analysis. IC_50_, inhibitory concentration 50%. *P < 0.1, **P < 0.01, ***P < 0.001 and ****P < 0.0001.

In addition to the above findings, immunoinfiltration analysis was performed. The 15 immune cells were significantly different in the high-risk group (HRG) and low-risk group (LRG) samples, including regulatory T cells (Tregs) (*P* = 3.18e-16), resting mast cells (*P* = 7.43e-10), and M0 macrophages (*P* = 4.59e-8) (**Figures 7A, B**). Notably, there was a significant positive correlation between ATP1A1 and M1 macrophages (P = 0.001) (**Figure 7C**). These outcomes suggested that ATP1A1 might contribute to disease development by controlling particular immune cells, facilitating a more comprehensive understanding of the core pathogenic pathways.

### 3.8 Differences in biological pathways between two risk cohorts were specified

A total of 14 pathways were significantly different between the two risk cohorts according to the threshold of *P* < 0.05. Among them, “complement and coagulation cascades,” “cytokine–cytokine receptor interaction,” and “olfactory transduction” were significantly up-regulated in the high-risk cohort, whereas “propanoate metabolism” and “valine, leucine, and isoleucine degradation” were up-regulated in the low-risk cohort (**Figure 7D**). Additionally, the GSVA results showed that nine signaling pathways such as “KRAS signaling DN,” “IL6/JAK/STAT3 signaling,” and “inflammatory response” were significantly activated in the high-risk cohort, whereas 21 signaling pathways such as “TGF-β signaling,” “PI3K/AKT/mTOR signaling,” and “NOTCH signaling” were significantly inhibited in the high-risk cohort (**Figure 7E**).

### 3.9 The IC_50_ values of 47 drugs were significantly differed between the two risk cohorts

The results of Spearman correlation analysis revealed that the IC_50_ values of 47 drugs were significantly correlated with risk score (**Figure 7F**) and were significantly different in the two risk cohorts (*P* < 0.05). After sorting the P values from small to large, the top 15 drugs were selected to show their differences between the two risk cohorts (**Figure 7G**). The box plot results showed that the IC_50_ values of the remaining drugs were significantly lower at the high-risk cohort, except for AS601245, Embelin, Epothilone.B, and FH535, suggesting that these drugs might be more likely to affect patients in the high-risk cohort.

### 3.10 Prognostic gene expression was assessed in two datasets

**Figure 8:**
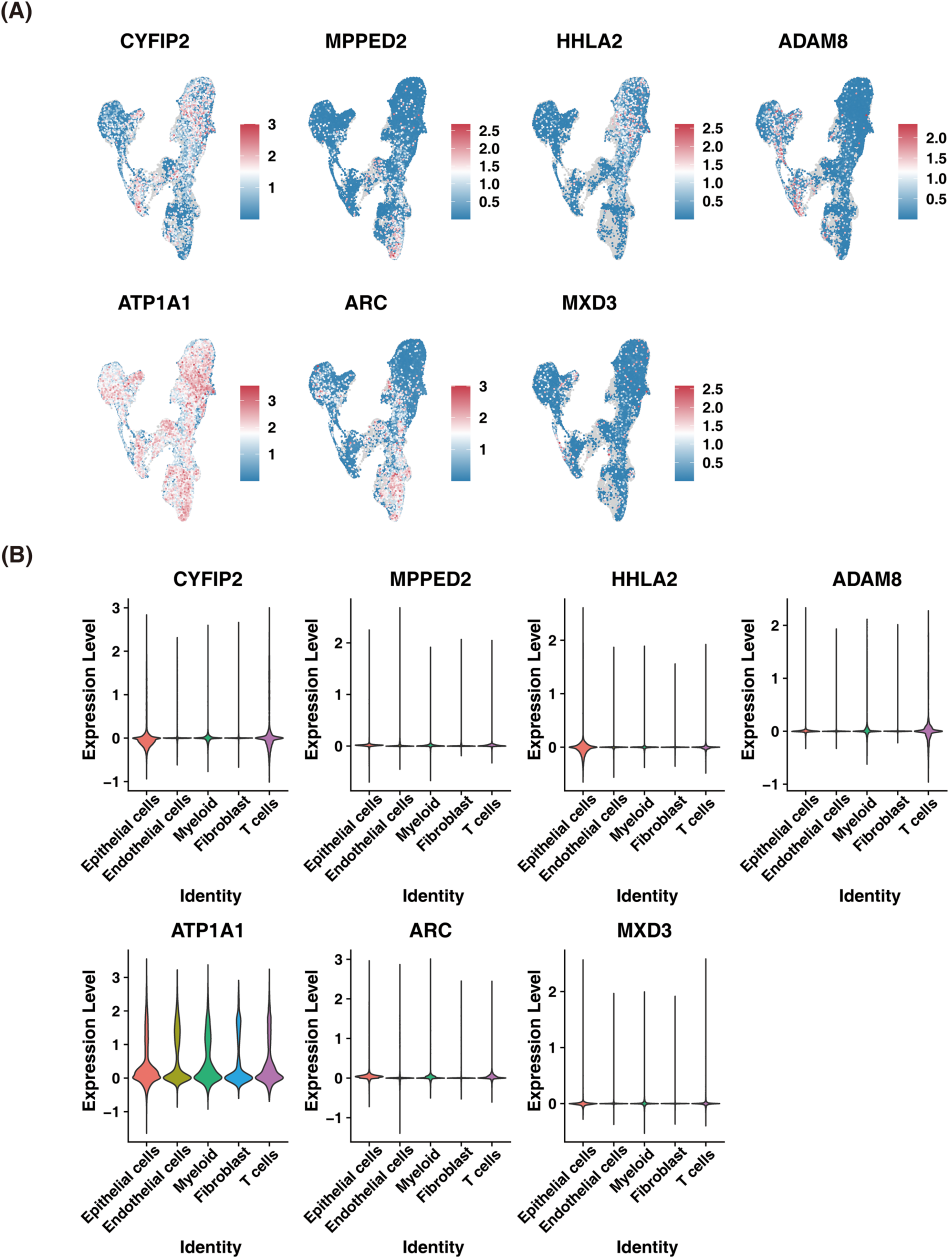
Prognostic gene expression was assessed in two datasets. **(A)** UMAP profile of prognostic genes expressed in different cell types. **(B)** Violin plot of prognostic genes expressed in different cell types. UMAP, uniform manifold approximation and projection.

The prognostic gene expression in epithelial cells, cell types annotated in the GSE156632 dataset, and each sample within the GSE175540 dataset were assessed. The results demonstrated that ATP1A1 was more highly expressed in epithelial cells (**Figure 8A**) and other cells annotated in the GSE156632 dataset (**Figure 8B**). Furthermore, the expression of these prognostic genes in each sample within the GSE175540 dataset is displayed in **Supplementary Figure 5**. Additionally, the expression of prognostic genes in these immune cells within each sample from the GSE175540 dataset is depicted in **Supplementary Figure 6**. The results revealed that prognostic genes were highly expressed in monocytic lineage, neutrophils, T cells, and fibroblasts.

### 3.11 ATP1A1 inhibited migration and tube formation of HUVECs

**Figure 9:**
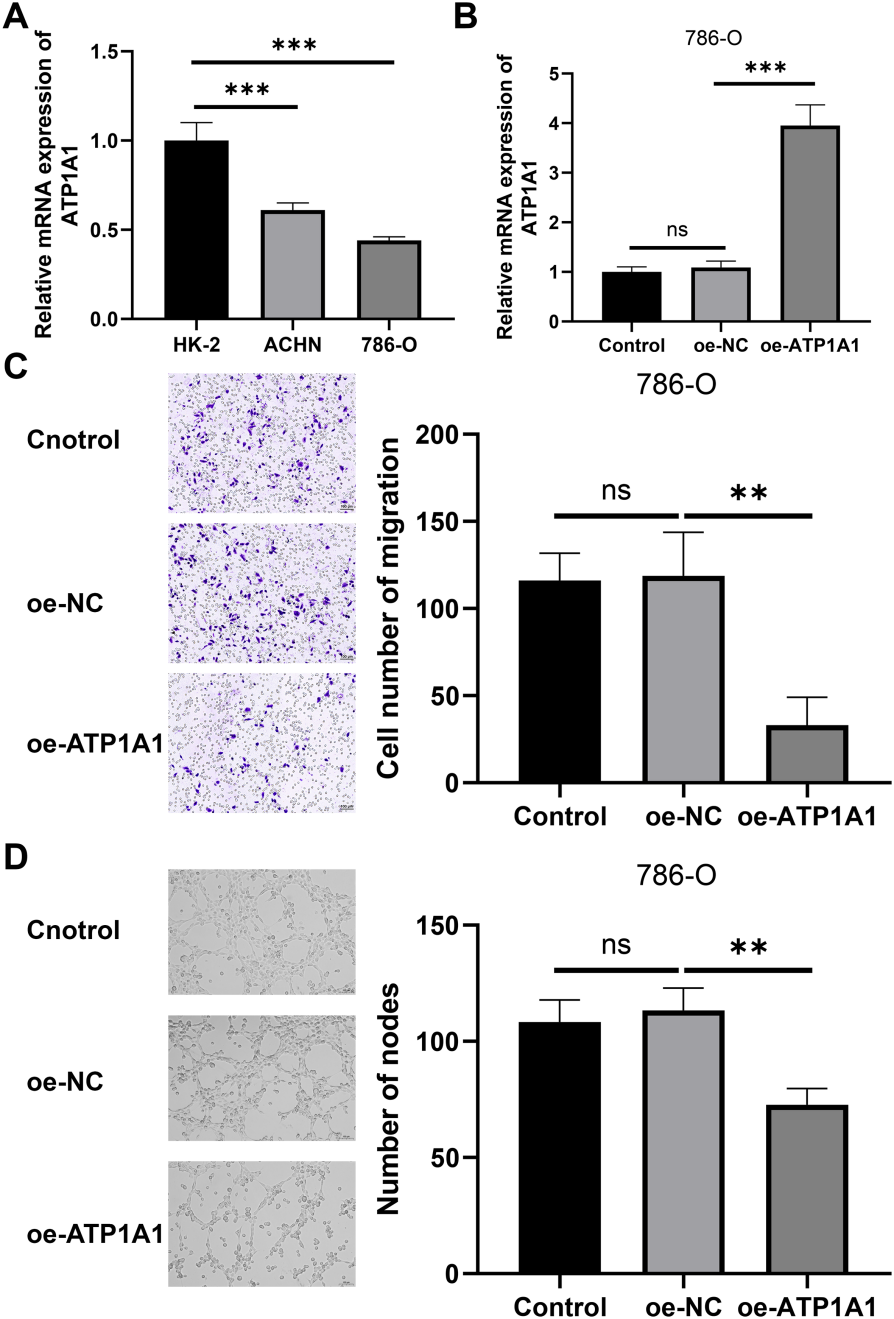
ATP1A1 inhibited migration and tube formation of HUVECs. **(A)** The mRNA expression of ATP1A1 in HK-2, ACHN, and 786-O cells was detected by qRT-PCR. **(B)** qRT-PCR was used to verify the overexpression efficiency of ATP1A1 in 786-O cells. **(C)** Transwell assay was used to verify the effect of ATP1A1 on HUVEC migration ability. The scale bar represents 100 μm. **(D)** The tube formation assay was conducted using HUVECs treated with different groups of 786-O cells. The scale bar represents 100 μm (All n=3). ns represents no significantly difference, **P < 0.01 and ***P < 0.001.

The above research suggests that ATP1A1 plays a crucial role in regulating endothelial cells and macrophages. Therefore, we conducted further studies on the effects of ATP1A1 on endothelial cells and macrophages. We utilized quantitative reverse transcription polymerase chain reaction (qRT-PCR) to measure mRNA levels of ATP1A1 in HK-2, ACHN, and 786-O cell lines. As a result, ATP1A1 expression was reduced in two ccRCC cell lines compared with HK-2 cells (All P< 0.01, **Figure 9A**). We chose the 786-O cell line with a lower expression level for the subsequent experiments. Subsequently, the ATP1A1 overexpression cell line (786-O) was constructed by plasmid vector, and the ATP1A1 mRNA expression was up-regulated as determined by quantitative polymerase chain reaction (qPCR) (*P* < 0.001, **Figure 9B**). Subsequently, HUVECs were cocultured with different CM from 786-O cells (Control, oe-NC, or oe-ATP1A1). Transwell assay demonstrated that the migration ability of HUVECs in the oe-ATP1A1 group was markedly down-regulated (**Figure 9C**). Tube formation assay demonstrated that the number of nodes of HUVECs in the oe-ATP1A1 group was markedly down-regulated (**Figure 9D**). The results of cell experiments confirmed that ATP1A1 could inhibit migration and tube formation of HUVECs via ccRCC.

### 3.12 ATP1A1 promoted macrophage M1 polarization

**Figure 10:**
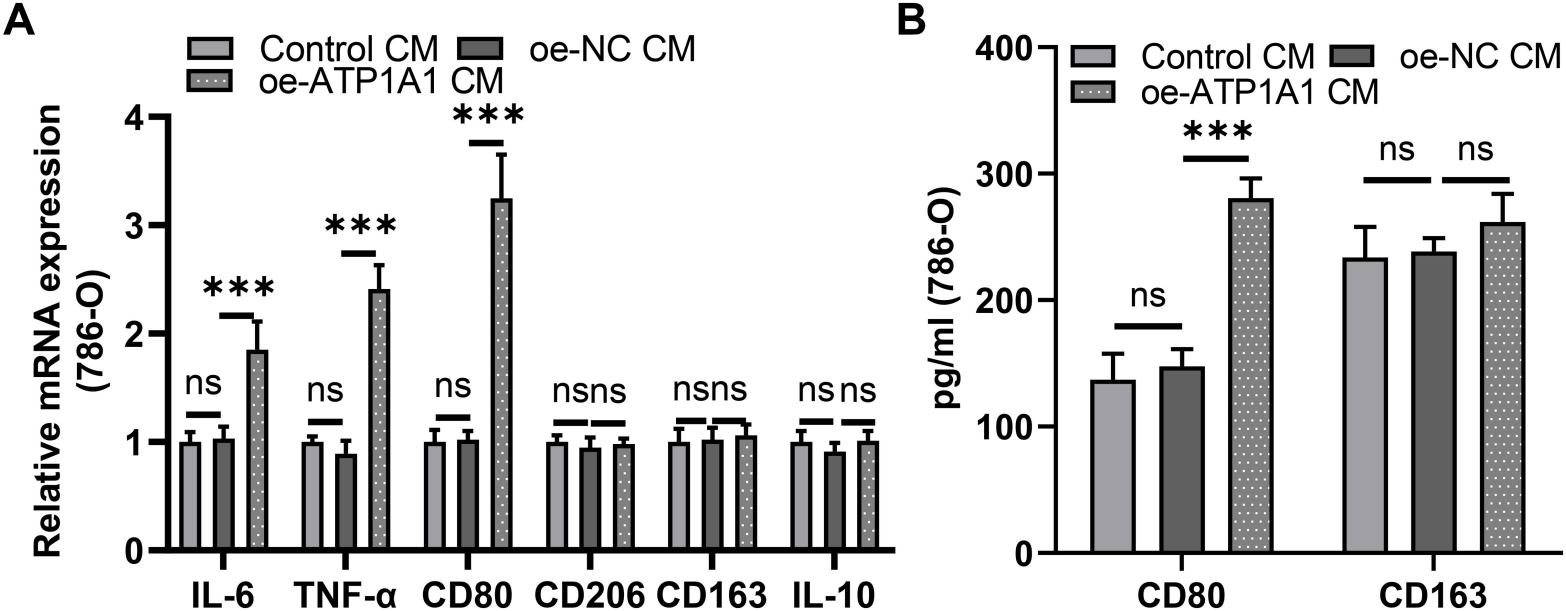
ATP1A1 promoted macrophage M1 polarization. **(A)** The mRNA levels of M1 (IL-6, TNF-a, and CD80) and M2 (CD206, CD163, and IL-10) markers in macrophages treated with 786-O-cell media of different treatment groups were detected by qRT-PCR. **(B)** The expression levels of CD80 and CD163 in macrophages treated with 786-O-cell media of different treatment groups were detected by ELISA (All n=3). ns represents no significantly difference and ***P < 0.001.

In addition to the activity of endothelial cells, we also investigated the effect of ccRCC cells with abnormal expression of ATP1A1 on macrophage polarization. Macrophages cocultured with CM from overexpressed ATP1A1 (786-O) cells significantly increased M1 markers (IL-6, TNF-α, and CD80) expression. Compared with the expression of M2 markers (CD206, CD163, and IL-10) in the control group, the expression of the M2 marker in the oe-ATP1A1 group was no significant difference (**Figure 10A**). In addition, the levels of macrophage polarization markers were analyzed using ELISA. Overexpression of ATP1A1 enhanced CD80 expression. However, it did not alter the expression of CD163 (**Figure 10B**). The above results suggested that ATP1A1 promoted M1 polarization of macrophages in ccRCC without affecting M2 polarization of macrophages.

## 4 Discussion

Despite benefiting from the tyrosine kinase inhibitor, antiangiogenesis drugs, and immune checkpoint inhibitors, patients with advanced ccRCC still face a dismal outcome, with a 5-year survival rate of less than 10% (32). This study identified prognostic genes associated with epithelial cells and established a prognostic model for ccRCC using comprehensive data from scRNA-seq and ST, facilitating prognosis prediction and improving clinical decision-making. Through a series of bioinformatics analyses, we found that epithelial cells are the most prevalent cell type in ccRCC. Spatial transcriptomic analysis further reveals significant spatial heterogeneity of epithelial cells in ccRCC. Epithelial cells are predominantly localized in the tumor core region, whereas at the tumor margin, they exhibit more frequent interactions with immune and stromal cells (33–35). Studies demonstrate that epithelial cells modulate the activity of immune and stromal cells within the tumor microenvironment through the secretion of cytokines and growth factors such as TGF-β and VEGF. These factors not only promote tumor cell proliferation and invasion but also suppress the antitumor activity of immune cells (36–38). These findings provide novel insights for early diagnosis and treatment of ccRCC. For instance, TGF-β expression levels may serve as an indicator for assessing tumor aggressiveness and prognosis, whereas inhibitors targeting the TGF-β signaling pathway could represent the potential therapeutic target. The highly abundant pathways related to epithelial cells include organic anion transport, degradation of GABA (Gamma-aminobutyric acid), alanine metabolism, and proline catabolism. Moreover, our findings indicated that epithelial cells interact with four other cell types, with particularly strong interactions observed between epithelial and myeloid cells. The MIF-CD74+CXCR4 axis may function as a crucial mediator, forming a bridge between epithelial and myeloid cells. MIF is a multifunctional immune regulatory factor that is overexpressed in many types of cancer and has complex connections with the marked biological characteristics of tumors and the regulation of tumor immunity (39). CD74 and CXCR4 are MIF receptors that interact with MIF to activate a variety of downstream signaling cascades, such as ERK and PI3K/Akt, which promote cell survival, proliferation, migration, angiogenesis, and immune evasion (40). A study on papillary thyroid carcinoma (PTC) suggested that malignant epithelial cells may transmit signals through MIF-(CD74+CXCR4) to macrophages, thereby suppressing their immune activity and promoting lymph node metastasis (41). Pseudotime analysis revealed that the occurrence and development of ccRCC are possibly associated with alterations in the differentiation trajectory or state of epithelial cells.

In this study, seven prognostic genes, namely, CYFIP2, MPPED2, HHLA2, ADAM8, ATP1A1, ARC, and MXD3, were screened out. Cytoplasmic fragile X mental retardation 1 (FMR)-interacting protein 2 (CYFIP2) shows low expression levels in ccRCC—strongly regulated by DNA methylation—that significantly correlate with poor survival prognosis and immune infiltration (42). The metallophosphoesterase-domain-containing protein 2 (MPPED2), frequently downregulated in tumors via promoter methylation, acts as a tumor suppressor by inhibiting proliferation and migration (43); our study is the first to report its prognostic role in ccRCC. Human endogenous retrovirus-H long terminal repeat-associated 2 (HHLA2) is an emerging immune checkpoint molecule that is widely expressed in tumors, but the prognostic value of its high expression in ccRCC remains inconclusive (44, 45). As a member of the disintegrin and metalloprotease (ADAM) family, ADAM8 has been shown to promote malignant behaviors such as proliferation and invasion in ccRCC cells, and the antitumor effects of the PD1 antibody could be potentiated by ADAM8 knockout (46). ATPase Na+/K+ transporting subunit alpha 1 (ATP1A1) is recognized as a prognostic factor in ccRCC, as evidenced by its low expression negatively correlating with the T stage (47). Cytological experiments have also revealed that ATP1A1 is connected to tumor cell growth, migration, and invasion (48). Activity-regulated cytoskeleton-associated protein (ARC) strengthened the tumor immunological milieu through dendritic cell migration and subsequent T-cell recruitment, thereby potentially improving the therapeutic efficacy of DC vaccines (49). MAX dimerization protein 3 (MXD3), a negative transcriptional regulator, is associated with poor outcomes and may shape the immune landscape in ccRCC (50). Collectively, these genes influence ccRCC progression, prognosis, and immunotherapy response through diverse mechanisms.

Based on these seven prognostic genes, this study constructed a corresponding risk prediction model. In the TCGA-ccRCC training cohort, the model demonstrated certain prognostic stratification efficacy. The risk score effectively distinguished survival differences between high- and low-risk patients. Furthermore, ROC curve analysis showed that it achieved significant AUC values for predicting 1-, 3-, and 5-year overall survival, indicating the potential assessment value of this gene signature for ccRCC patient prognosis.

GSEA revealed that the high-risk group was primarily associated with immune and inflammatory responses (e.g., the complement and coagulation cascade pathway). The hypercoagulable state induced by coagulation system activation in malignant tumors can lead to common thromboembolic complications such as renal vein or inferior vena cava thrombosis in RCC, contributing to worse survival (51). In addition, the coagulation process and various complement components intricately interact with the tumor microenvironment, influencing the recruitment and activation of multiple immune cells (52, 53). Another pathway that was significantly activated in high-risk populations is cytokine–cytokine receptor interaction. Cytokines are engaged in the regulation of numerous cellular processes that either promote or inhibit tumors (54). The GSVA demonstrated that the most enriched pathways include heme metabolism, TGF-β signaling, and the downregulation of KRAS signaling. Dysregulated heme metabolism in cancer exhibits a dual role: High concentrations of heme support carcinogenic transformation by generating reactive oxygen species (ROS) and cytotoxicity, downregulating P53 expression, modulating immune cell function, and inducing chronic inflammation (55), whereas heme-mediated ROS accumulation can also suppress tumors through ferroptosis (55). The TGF-β signaling shows stage-specific roles: It exerts tumor-suppressive effects in the early stages and promotes tumor invasion and metastasis in the later stages (56). A recent study found that the TGFβ signaling pathway genes were relatively underexpressed in RCC but overexpressed in the tumor microenvironment (TME). This imbalance led to the loss of the antiproliferative and pro-apoptotic abilities of the cytokine, as well as immune suppression in the tumor surrounding space and inhibition of non-tumor cell proliferation, which might determine the tumor’s invasiveness and metastatic potential (57). Evidence has suggested that inhibiting the TGF-β pathway could reduce ccRCC invasion (58). Overall, factors related to inflammation, immunity, or cytokines may underlie the development and prognosis of ccRCC, which requires further in-depth research.

ccRCC is a highly immune-infiltrated malignancy where diverse immune cells within the tumor microenvironment can either promote or suppress immune responses (59). The composition of immune infiltration and tumor-immune cell interactions critically influence disease progression and response to immunotherapy (59). Our analysis preliminarily reveals potential differences in the immune landscape between different risk groups. According to the available data, high-risk patients may exhibit an increasing trend in the infiltration of Tregs and M0 macrophages, whereas low-risk patients may be characterized by an accumulation of resting mast cells. As key cells in both innate and adaptive immunity, macrophages are recruited into tumors as undifferentiated M0 macrophages and can polarize into M1 or M2 phenotypes (60). M1 macrophages exert antitumor effects through direct cytotoxicity and activation of adaptive immunity (60). In RCC, M1 infiltration is associated with prolonged survival, whereas M2 infiltration is linked to poor prognosis (61). Infiltrating mast cells have been reported to participate in angiogenesis in RCC, potentially mediated by the PI3K-AKT-GSK-3β-AM axis (62). Based on the observed accumulation of resting mast cells in the low-risk group in our study, we tentatively hypothesize that, compared with activated mast cells, resting mast cells might inhibit tumor progression, potentially by reducing the release of pro-angiogenic factors. This may thus be associated with a more favorable prognosis. This hypothesis, however, requires further experimental validation.

Among the 15 drugs with significant differences in IC_50_ values, AS601245, Embelin, Epothilone B, and FH535 may be more advantageous for patients in the high-risk group, whereas patients with low-risk ccRCC are more likely to benefit from the other drugs. Although most of these drugs have not yet been applied in the treatment of ccRCC, they have presented encouraging anticancer activity in previous studies. Among them, the plant-derived natural benzoquinone compound Embelin displays antitumor effects by targeting key signaling pathways and inducing apoptosis and autophagic cell death (63). Moreover, it can enhance the anti-proliferative and pro-apoptotic effects of the tyrosine kinase inhibitor Axitinib by blocking the HIF pathway (64). The clinically commonly used small-molecule receptor tyrosine kinase inhibitor Sunitinib inhibits tumor angiogenesis, blocks tumor growth and metastasis, and modulates the tumor microenvironment by targeting the vascular endothelial growth factor (VEGF) and platelet-derived growth factor (PDGF) (65). Due to its significant therapeutic advantages, it is listed as a first-line treatment for advanced and metastatic ccRCC (66). The results of this study suggest that Sunitinib could provide a suitable treatment option for low-risk patients. In conclusion, our findings preliminarily suggest the potential value of the aforementioned drugs in ccRCC. However, the actual efficacy, appropriate patient populations, and mechanisms of action of these drugs still require further validation through large-scale clinical studies or additional *in vitro* functional experiments, in order to provide more reliable references for the precise treatment of ccRCC.

Improvement of ccRCC treatment requires delving into the characteristics and underlying mechanisms of the tumor immune microenvironment. Through investigating the prognostic gene profiling of epithelial cells, we found that ATP1A1 was highly expressed in epithelial cells, endothelial cells, myeloid cells, fibroblasts, and T cells. ATP1A1 often functions as a signaling molecule to activate intracellular signaling cascades (67). ATP1A1 has a wide range of effects on the biological behaviors of epithelial cells, such as cell proliferation and apoptosis, adhesion and migration, and intercellular contact and communication, implying a malignant phenotype of ccRCC cells (68, 69). In addition, this subunit enhances immune cell migration and adhesion by binding to cardiotonic steroids, ultimately promoting renal inflammation (70). Although ccRCC is classified as highly immunogenic due to the extensive infiltration of myeloid cells and lymphocytes, M2-like tumor-associated macrophages (TAMs), exhausted CD8^+^ T cells, and cancer-associated fibroblasts (CAFs) drive tumor progression and immune suppression, which are associated with an unsatisfactory prognosis for immune checkpoint inhibitor therapy (71). Apart from ATP1A1, studies on other prognostic genes are demonstrated.

Among these genes, ATP1A1 was noted for its dual roles in immune microenvironment modulation and vascular regulation. Our analysis revealed a positive correlation between ATP1A1 expression and M1 macrophage infiltration, suggesting a potential role in macrophage polarization. Coculture with ATP1A1-overexpressing ccRCC cells selectively upregulated M1 markers without altering M2 expression, confirming that ATP1A1 amplifies antitumor responses through M1 phenotype differentiation. Meanwhile, we observed significant downregulation of ATP1A1 in ccRCC cell lines. Notably, ATP1A1 overexpression demonstrated potent anti-angiogenic activity, specifically impairing both endothelial migration and tube formation capacity. These findings indicate that ATP1A1 downregulation may foster a permissive microenvironment for tumor progression by suppressing M1 macrophage-mediated antitumor immunity and promoting angiogenesis, highlighting its potential as a therapeutic target and prognostic biomarker in ccRCC.

This study, for the first time, developed a risk model containing seven prognostic genes identified in ccRCC and further assessed the prognostic value through an integrated analysis of scRNA-seq and ST data from ccRCC. Furthermore, we systematically mapped the cell-type-specific expression profiles of prognostic genes, with particular emphasis on ATP1A1’s pivotal roles in immunomodulation and vascular biology. This work establishes a framework for utilizing cell-specific molecular signatures to guide precision cancer therapeutics. However, our research still has some limitations. On the one hand, the mechanisms through which these prognostic genes influence ccRCC heterogeneity, along with their potential for clinical application, require further in-depth exploration. On the other hand, the functional validation of ATP1A1 has clear limitations. Specifically, the functional assays were performed only in the 786-O ccRCC cell line using an overexpression model, without parallel validation in other ccRCC cell lines such as ACHN, nor complementary analyses with gene knockdown or knockout models. These shortcomings make it difficult to exclude biases arising from cell line–specific genetic backgrounds and to comprehensively characterize the functional effects of ATP1A1. In addition, both the angiogenesis and macrophage polarization assays relied on CM derived from 786-O cells. Given the complex composition of CM—which may contain non-specific metabolites or signaling molecules in addition to the downstream secretory factors regulated by ATP1A1—it is difficult to rule out interference from these confounding components. Without direct experimental evidence to elucidate the underlying mechanism, the effects can only be inferred to result from alterations in the cellular secretome, thereby diminishing the strength of the mechanistic interpretation. To overcome these limitations, future work will focus on three aspects. First, ATP1A1 knockdown or knockout experiments will be conducted in ACHN and other ccRCC cell lines to compare functional outcomes across different cellular contexts and expression levels, thereby validating the generalizability of the conclusions. Second, secretome profiling will be performed on conditioned media from ATP1A1-overexpressing and control cells to identify key secretory factors, followed by rescue experiments using factor addition or neutralization to clarify their mediating roles in angiogenesis and macrophage polarization. Third, the activation status of relevant downstream signaling pathways will be evaluated in combination with co-immunoprecipitation assays to identify direct interaction partners of ATP1A1, thereby providing direct evidence for its molecular mechanism. These studies will further strengthen the reliability and depth of our conclusions and offer more rigorous and translationally meaningful evidence for prognostic management and targeted therapy research in ccRCC.

## Data Availability

The datasets presented in this study can be found in online repositories. The names of the repository/repositories and accession number(s) can be found in the article/**Supplementary Material**.

## Supplementary material

Supplementary Figure 1 Quality control and analysis of single-cell sequencing data. **(A)** Distribution of nFeature_RNA, nCount_RNA, percent.mt before quality control. **(B)** Distribution plot of nFeature_RNA, nCount_RNA, and percent.mt after quality control. **(C)** Screening for highly variable genes. **(D)** Plot of results for PCA-based dimensionality reduction analysis. PCA, principal component analysis.

Supplementary Figure 2 Distribution of nCount for each sample in the spatial transcriptome dataset.

Supplementary Figure 3 Distribution of nFeature for each sample in the spatial transcriptome dataset.

Supplementary Figure 4 Visualization of ST data subgroups’ locations. ST, spatial transcriptomics.

Supplementary Figure 5 Heatmap of prognostic gene expression in the GSE175540 dataset.

Supplementary Figure 6 Heatmap of prognostic gene expression in immune cells across samples in the GSE175540 dataset.
