# Supplementary material for: Prognostic gene screening and experimental validation in renal clear cell carcinoma based on spatial transcriptomics and single-cell sequencing
Source: Front Immunol. 2026 Jan 28;17:1699883. doi: 10.3389/fimmu.2026.1699883 (PMC12891194; doi:10.3389/fimmu.2026.1699883)
Supplement: Supplementary file 1 [file Table1.docx]

Supplementary Table S1: List of all primers used in qRT-PCR

| Primer | Forward sequence (5′-3′) | Reverse sequence (5′-3′) |
| --- | --- | --- |
| ATP1A1 | CTCTGATTCTCCAGCGACAGG | AACAGCTGCAGGCTCATACTT |
| IL-6 | TCGGTCCAGTTGCCTTCTC | GAGGTGAGTGGCTGTCTGTG |
| TNF-α | CCCTCACACTCAGATCATCTTCT | GCTACGACGTGGGCTACAG |
| CD80 | TCTCAGAAGTGGAGTCTTACCCT | GATTGGAGGGTGTTCCTGGG |
| CD206 | CCAAACGCCTTCATTTGCCA | ACCTTCCTTGCACCCTGATG |
| CD163 | CCGGGAGATGAATTCTTGCCT | GGTATCTTAAAGGCTCACTGGGT |
| IL-10 | AGGGCACCCAGTCTGAGAAC | TCTTCACTCTGCTGAAGGCAT |
| GAPDH | GCACCGTCAAGGCTGAGAAC | TGGTGAAGACGCCAGTGGA |
