# Supplementary figures and images for: Prognostic gene screening and experimental validation in renal clear cell carcinoma based on spatial transcriptomics and single-cell sequencing
Source: Front Immunol. 2026 Jan 28;17:1699883. doi: 10.3389/fimmu.2026.1699883 (PMC12891194; doi:10.3389/fimmu.2026.1699883)

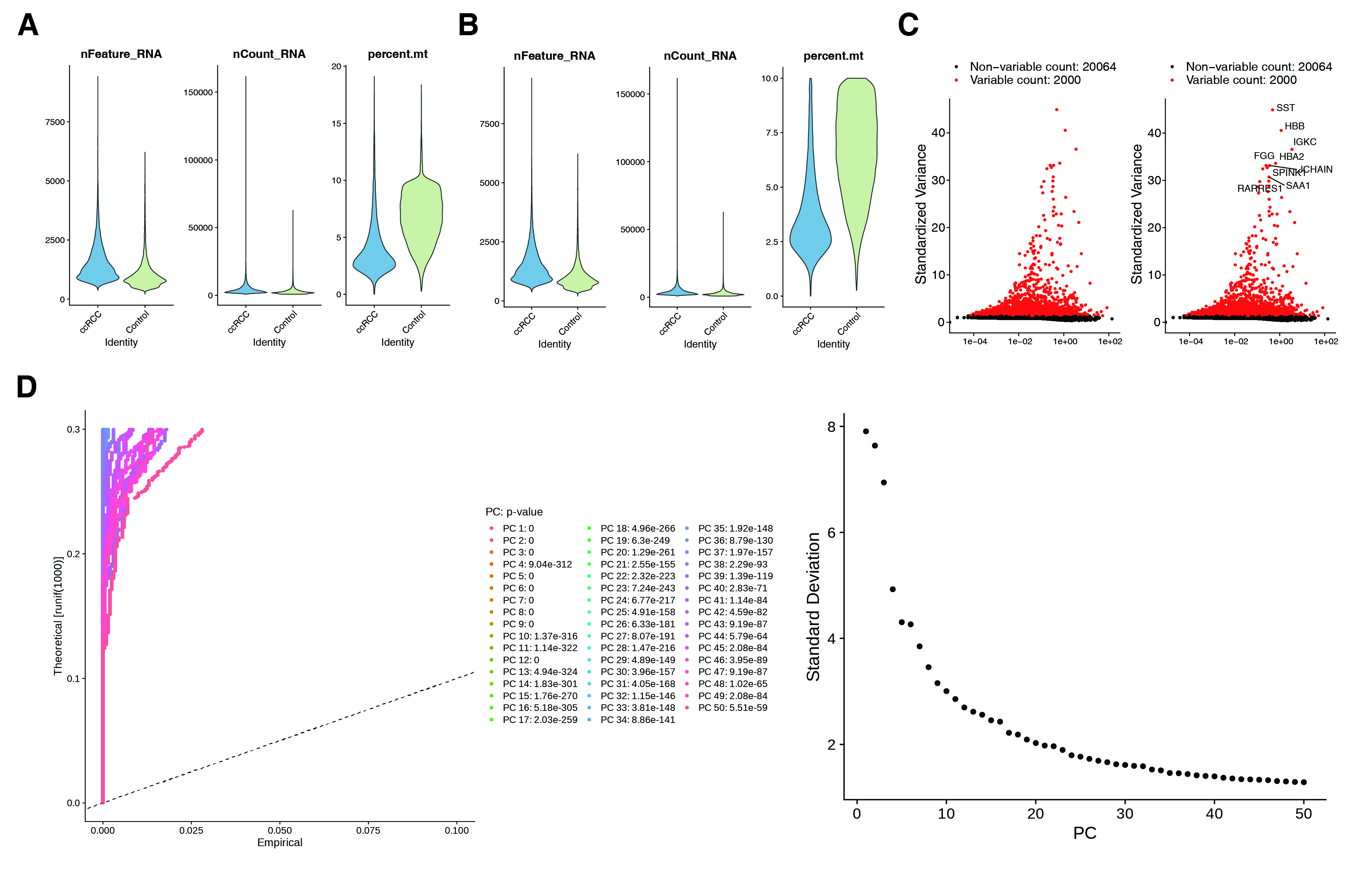

Supplement: Supplementary Figure 1 — Quality control and analysis of single-cell sequencing data. (A) Distribution of nFeature_RNA, nCount_RNA, percent.mt before quality control. (B) Distribution plot of nFeature_RNA, nCount_RNA, and percent.mt after quality control. (C) Screening for highly variable genes. (D) Plot of results for PCA-based dimensionality reduction analysis. PCA, principal component analysis. [file Image1.tif]

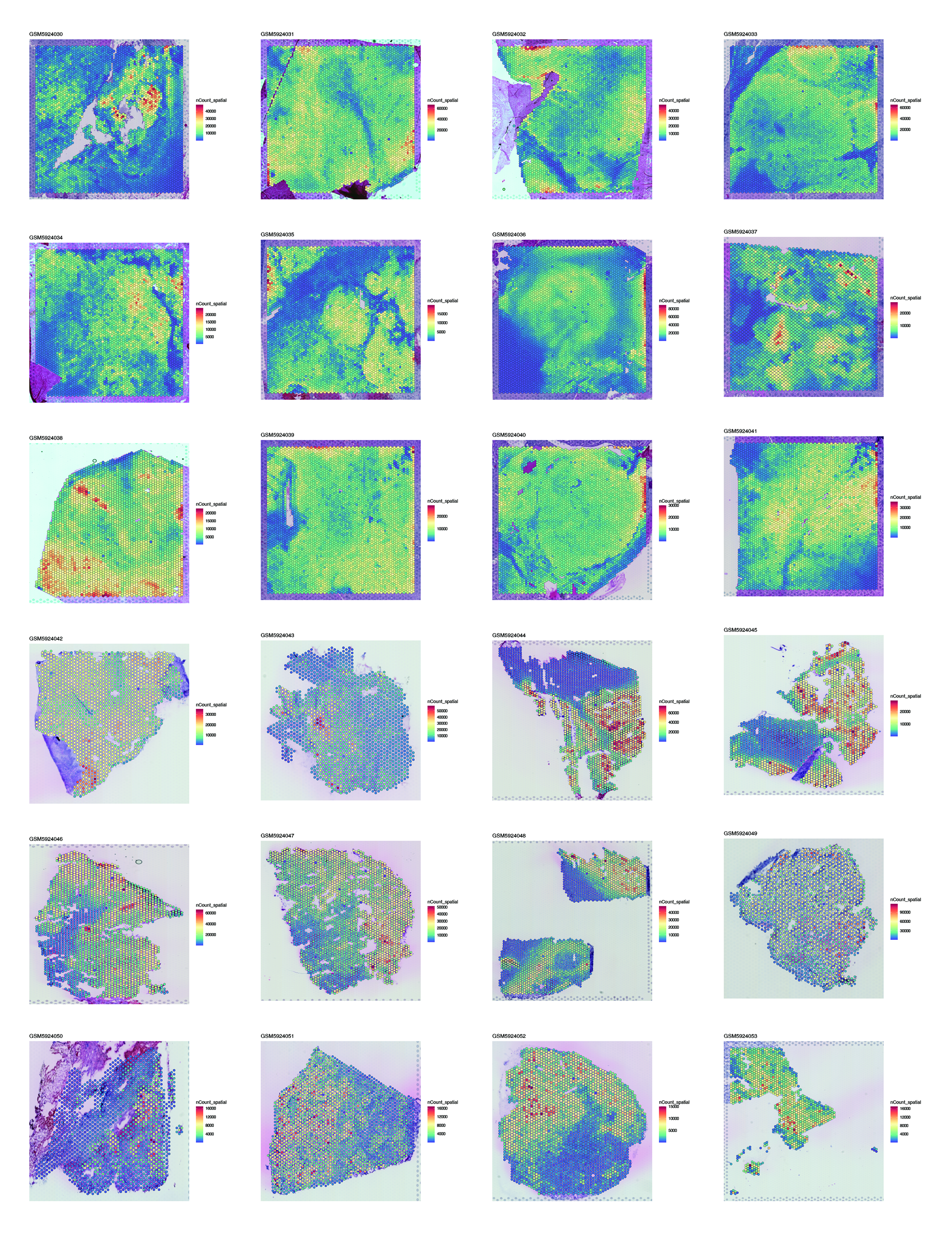

Supplement: Supplementary Figure 2 — Distribution of nCount for each sample in the spatial transcriptome dataset. [file Image2.tif]

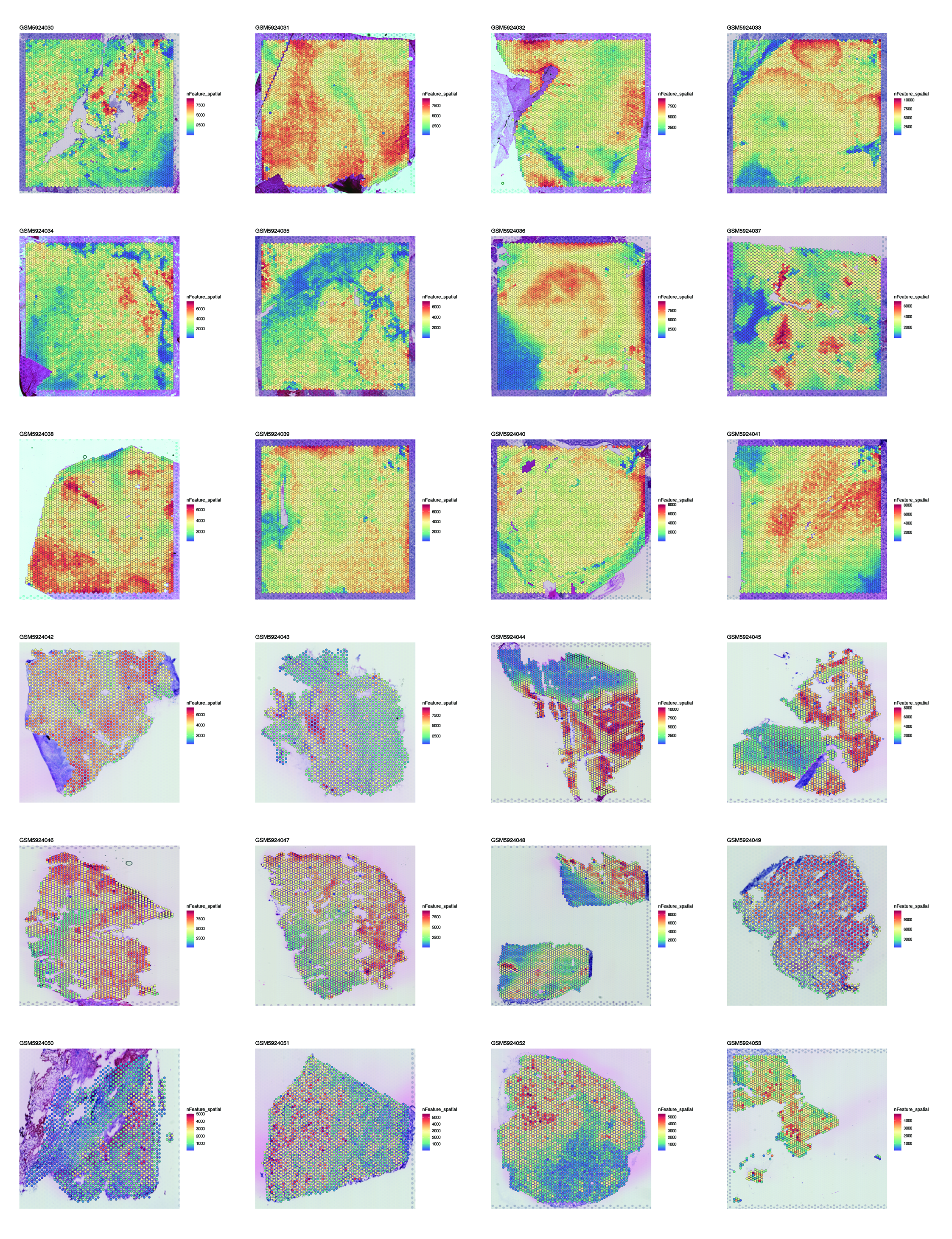

Supplement: Supplementary Figure 3 — Distribution of nFeature for each sample in the spatial transcriptome dataset. [file Image3.tif]

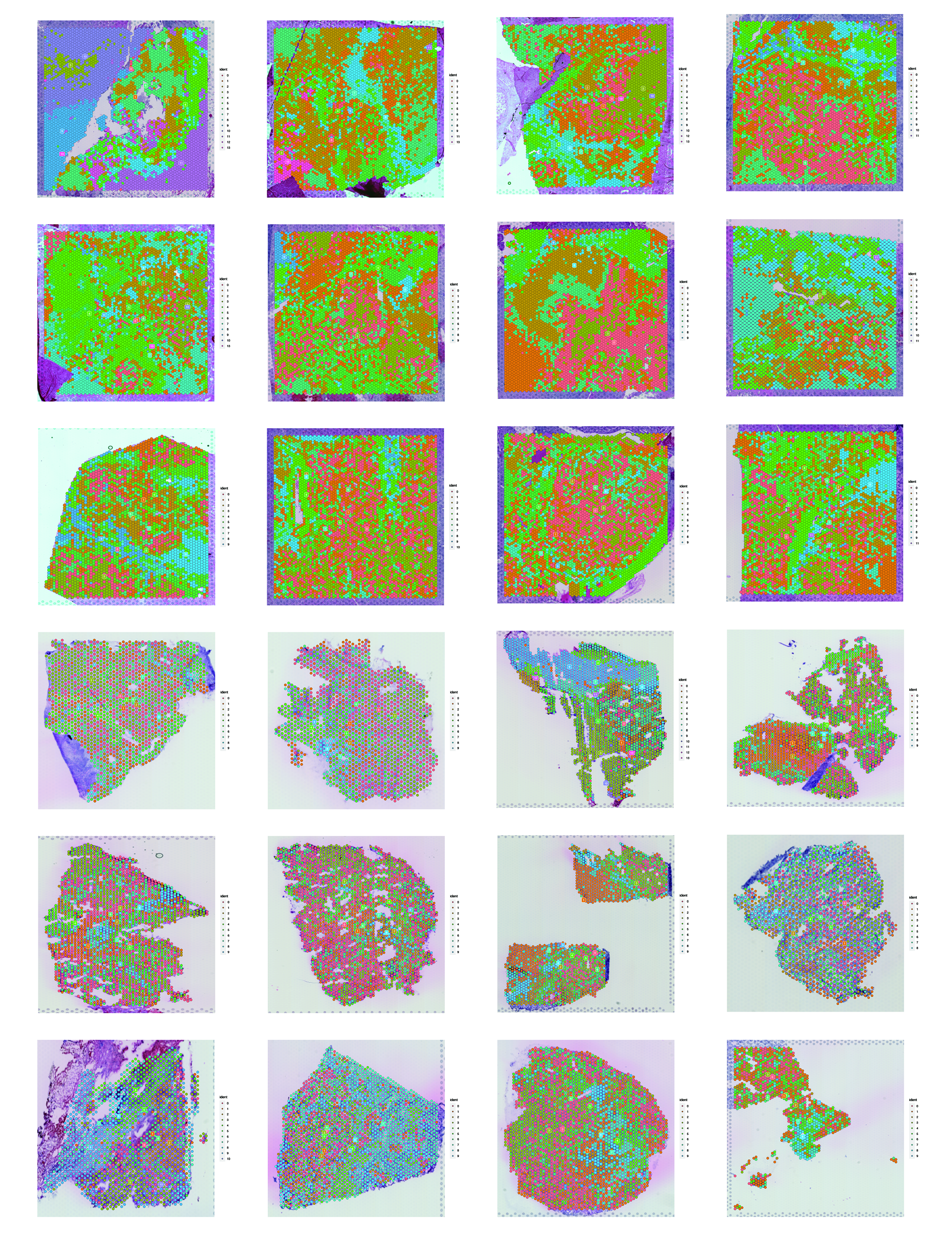

Supplement: Supplementary Figure 4 — Visualization of ST data subgroups’ locations. ST, spatial transcriptomics. [file Image4.tif]

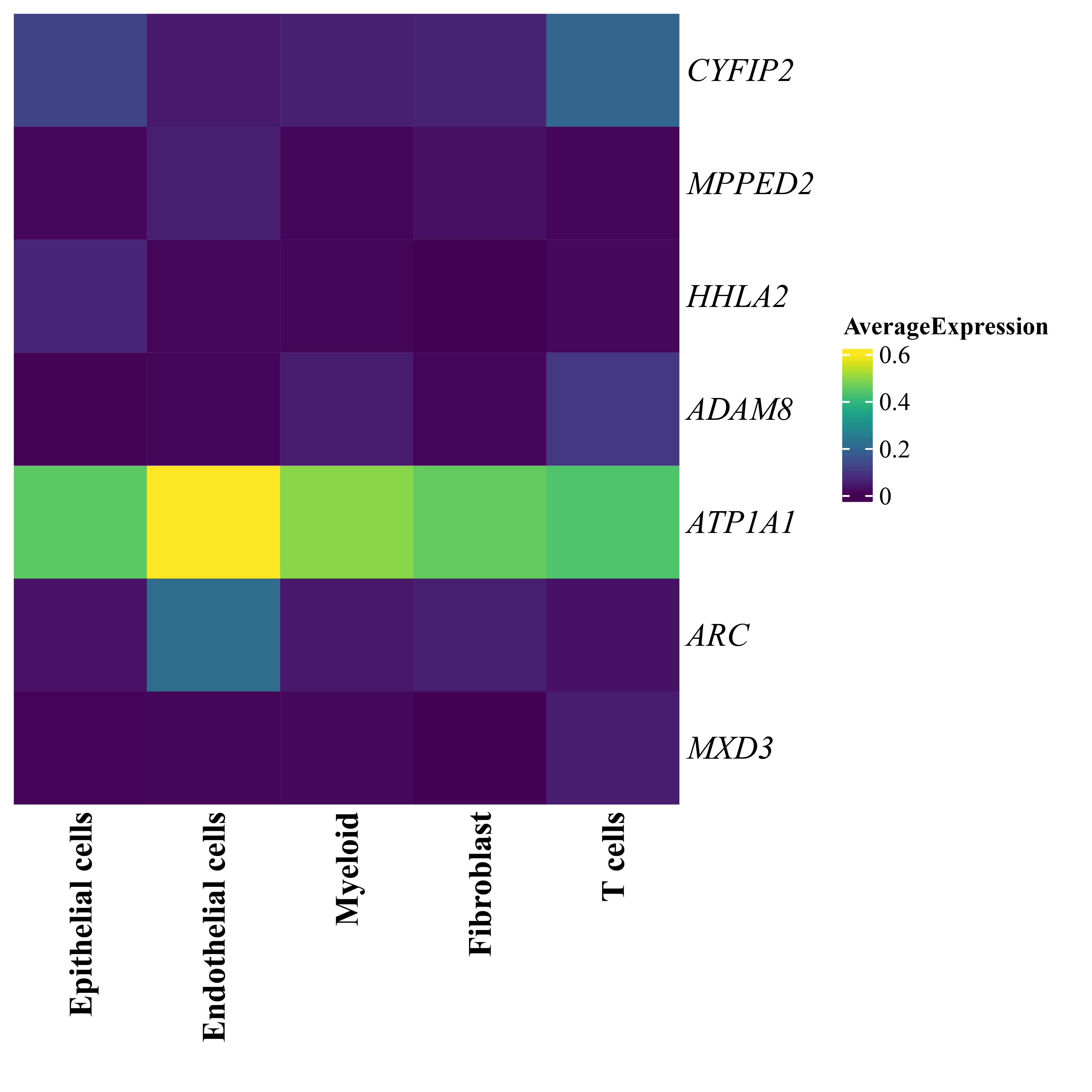

Supplement: Supplementary Figure 5 — Heatmap of prognostic gene expression in the GSE175540 dataset. [file Image5.tif]

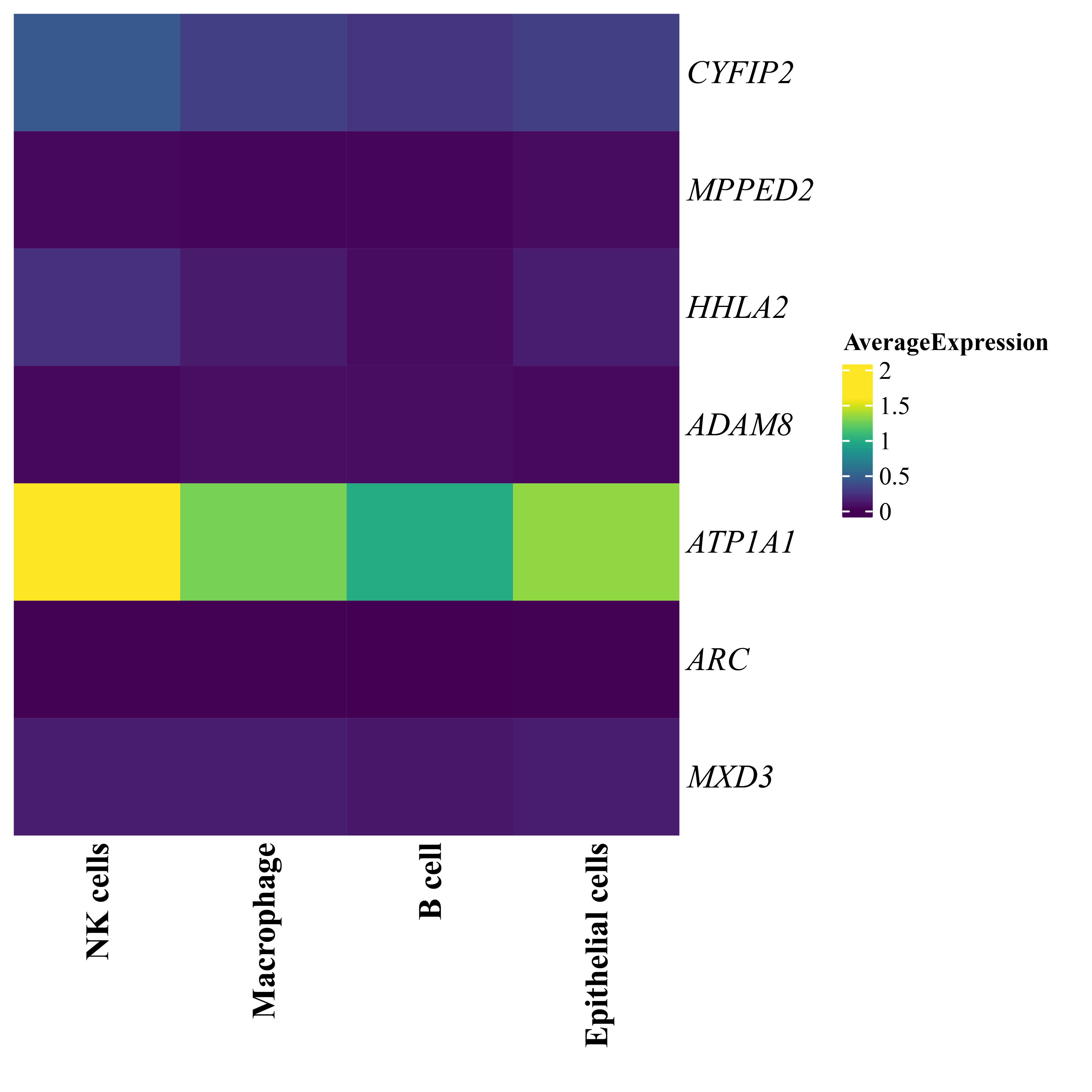

Supplement: Supplementary Figure 6 — Heatmap of prognostic gene expression in immune cells across samples in the GSE175540 dataset. [file Image6.tif]
